# Ingestion of ‘whole cell’ or ‘split cell’ *Chlorella* sp., *Arthrospira* sp., and milk protein show divergent postprandial plasma amino acid responses with similar postprandial blood glucose control in humans

**DOI:** 10.3389/fnut.2024.1487778

**Published:** 2024-11-14

**Authors:** Ellen Williamson, Alistair J. Monteyne, Ino Van der Heijden, Doaa R. Abdelrahman, Andrew J. Murton, Benjamin Hankamer, Francis B. Stephens, Benjamin T. Wall

**Affiliations:** ^1^Nutritional Physiology Group, Public Health and Sport Sciences, The Medical School, The University of Exeter, Exeter, United Kingdom; ^2^Institute for Molecular Bioscience, The University of Queensland, Brisbane, QLD, Australia; ^3^Department of Surgery, University of Texas Medical Branch, Galveston, TX, United States; ^4^Sealy Centre on Aging, University of Texas Medical Branch, Galveston, TX, United States

**Keywords:** alternative protein, algal protein, microalgae protein, sustainable food, protein ingredient

## Abstract

**Introduction:**

Microalgae provide a sustainable basis for protein-rich food production. However, human data concerning microalgae ingestion, subsequent postprandial amino acid (AA) availability and associated metabolic responses are minimal.

**Objectives:**

We investigated ingesting *Arthrospira* sp. (spirulina;SPR), and *Chlorella* sp. (chlorella; in ‘whole cell’ [WCC] and ‘split cell’ [SCC] forms, the latter proposed to improve digestibility), compared with a high-quality animal derived protein source (milk; MLK).

**Subjects/methods:**

Ten participants (age; 21 ± 1y, BMI; 25 ± 1 kg·m^−2^) completed a randomised, crossover, double-blind study, partaking in 4 counterbalanced (for order) experimental visits. At each visit participants ingested SPR, WCC, SCC or MLK drinks containing 20 g protein and 75 g carbohydrate. Arterialised venous blood samples, indirect calorimetry and visual analogue scales were assessed postabsorptive, and throughout a 5 h postprandial period to measure AA, glucose, insulin and uric acid concentrations, whole-body energy expenditure and appetite scores, respectively.

**Results:**

Protein ingestion increased plasma AA concentrations (*p* < 0.001) to differing total postprandial total—and essential—AA availabilities; highest for MLK (86.6 ± 17.8 mmol·L^−1^) and SPR (84.9 ± 12.5 mmol·L^−1^), lowest for WCC (−4.1 ± 21.7 mmol·L^−1^; *p* < 0.05), with SCC (55.7 ± 11.2 mmol·L^−1^) marginally greater than WCC (*p* = 0.09). No differences (*p* > 0.05) were detected between conditions for postprandial glucose or insulin concentrations, whole-body energy expenditure or appetite scores, but serum uric acid concentrations increased (*p <* 0.05) following microalgae ingestion only.

**Conclusion:**

Our data imply that microalgae can present a bioavailable source of protein for human nutrition, however, challenges remain, requiring species selection and/or biomass processing to overcome.

## 1 Introduction

Environmental and ethical concerns surrounding contemporary dietary protein production (in particular animal-derived proteins) are driving interest in novel and sustainably produced protein sources (1, 2). By way of example, it has been projected that by 2035, 11% of the overall dietary protein market will be made up by alternative proteins (compared with 2% in 2020) (3, 4). A particularly promising group of alternative, non-animal protein sources are microalgae. Microalgae are aquatic photosynthetic microorganisms, and in the present work we also include photosynthetic cyanobacteria under the broader term of ‘microalgae’. Literature reviews of the suitability of microalgae as a food source (5–9) have highlighted that their cultivation systems offer more sustainable production of protein compared to terrestrial crops and traditional animal agriculture, based on their relatively low land, fresh water and fertiliser requirements, as well as low CO_2_ emissions (8). Crucially, although genetically diverse (10), multiple species of microalgae are naturally rich in protein, on par with animal- and plant-based protein-rich foods or concentrates (35–80% protein) (8, 11). Two species, *Arthrospira* sp. (commonly known as spirulina) and *Chlorella* sp. (referred to as chlorella), currently dominate the global market of microalgae in human nutrition, emerging from regionally traditional foods to modern micronutrient-rich supplements, typically consumed in small amounts (12–14). However, both species are also naturally high in protein (in some instances up to 70% of their dry weight) and, accordingly are now being considered for their potential to contribute to widespread human dietary protein requirements (8).

Dietary protein intake is vital to support human health. Following protein ingestion, amino acids absorbed into the circulation act as signal and substrate for the stimulation of whole-body and tissue-specific protein synthesis, required to maintain body proteostasis, as well as to partly fulfil daily energy requirements (15, 16). However, postprandial plasma amino acid kinetics are specific to a given protein source, determined by various factors, including protein content of the food, amino acid composition, digestibility and intestinal absorption (17). Generally, plant-derived protein sources are considered to be of lesser quality in terms of human requirements, than animal-derived proteins (18, 19), due to typically lower and less balanced essential amino acid compositions and/or inferior protein digestion and absorption kinetics (18, 20). Microalgae protein compositions are typically more similar to animal-derived proteins, in that they are protein rich and balanced in all the essential amino acids (i.e., no single amino acid deficiencies) (8, 20). However, human data investigating the effect of their ingestion on plasma amino acid availability are scarce and, as such, how they compare in terms of their protein bioavailability is largely unknown. Devi et al., 2018 (21) measured relative ileal digestibility of *Arthrospira platensis in vivo* in humans, using intrinsically labelled [13C]-*A. platensis*. They reported the average ileal digestibility of *A. platensis* derived essential amino acids as ~85%, comparable to milk proteins (22, 23). Similarly, our group have directly compared human plasma amino acid concentrations following the ingestion of spirulina and milk reporting them as comparable, while chlorella proteins were less bioavailable than both (24, 25).

A plausible explanation for these data are species differences in the cell wall type of spirulina and chlorella. Chlorella possesses a more robust cell wall structure, therefore likely less predisposed to human digestion (10, 26, 27). In support of a cell wall limitation, biomass processing steps (e.g., mechanically breaking the cell wall) have indicated improved protein digestibility. *In vitro* this is demonstrated by an increased release of soluble proteins following cell disruption (26, 28, 29). Rodent models generally concur, demonstrating that chlorella protein is better digested (assessed via ileal digestibility and/or faecal nitrogen) in cell disrupted vs. unprocessed biomass (30, 31), although no marked improvements are observed for spirulina, suggesting that monogastric digestive systems may be sufficient to digest spirulina regardless (32). However, in the absence of human data, it is currently unknown if cell wall disruption methods are also sufficient to improve human *in vivo* amino acid uptakes, with chlorella of most relevance.

For novel proteins to be considered for bulk human consumption, other aspects of postprandial metabolism should also be evaluated. For instance, dietary protein ingested within a mixed meal also plays a role in modulating postprandial glycaemia (33), and contributes considerably to dietary-induced thermogenesis (34) and feelings of satiety (35, 36). Additionally, single-cell protein-rich novel foods (i.e., microalgae) fall under the FAO/WHO/UNICEF Protein Advisory Group recommendation to limit dietary nucleic acid (DNA and RNA) load to 2 g·day^−1^ (37, 38). A constituent of nucleic acids are purines, which are metabolised in the liver forming uric acid. Uric acid, if elevated in blood serum, represents a risk factor for various cardio-metabolic diseases (37, 39). Microalgae are naturally rich in nucleic acids (~6.6 g·100 g^−1^ dry weight), dependent on growth phase, and taxonomy (e.g., chlorophyta: ~5.9 g·100 g^−1^ dry weight, cyanobacteria: ~9.5 g·100 g^−1^ dry weight) (40) which raises concerns regarding hyperuricemia. Specifically, purine contents of spirulina (1076 mg·100 g^−1^) and chlorella (3182 mg·100 g^−1^) reported in the literature are far in excess of more traditional foods such as cereals and beans (< 50 mg·100 g^−1^), eggs and dairy products (<13 mg·100 g^−1^), and a variety of raw meats (69–285 mg·100 g^−1^) (41). These aspects of acute postprandial metabolism concerned with bulk microalgae ingestion have not been comprehensively considered in human studies, and such data are crucial to establish whether unprocessed microalgae are suitable to support human nutrition.

The primary aim of the present study was to characterise the postprandial plasma amino acid response following ingestion of isonitrogenous boluses of commercially available spirulina (SPR) and chlorella compared with milk protein as a high-quality reference protein source (42). We investigated chlorella in both ‘whole cell’ [WCC; referring to the cells being intact (43)] and ‘split cell’ [SCC; referring to the cell walls being broken (44)] preparations to also assess the influence of the cell wall on postprandial plasma amino acid availability. We hypothesised that SCC would have a higher plasma amino acid availability comparable to WCC, but that both chlorella conditions would be lower than milk and spirulina. A secondary goal was to assess the impact of the ingested proteins on serum uric acid concentrations, whole-body energy expenditure, subjective appetite and postprandial glycaemic control; to achieve the latter carbohydrate was co-ingested with each protein bolus (45, 46).

## 2 Methods

### 2.1 Participants

Participant recruitment and data collection were carried out in the Nutritional Physiology Research Unit at the University of Exeter between August 2021 and May 2022. This study was approved by the Sport and Health Sciences Ethics Committee of the University of Exeter (21–07-14-A-01) in accordance with the Declaration of Helsinki and is registered at ClinicalTrials.Gov (NCT05401591). Experimental procedures, potential risks, and the purpose of the study were explained to the participants prior to obtaining informed written consent. Before their inclusion in the study, participants attended a screening session which consisted of assessments of body mass, height, blood pressure, body composition (BodPod, Life Measurement, Inc.), and the completion of a routine medical screening questionnaire. Participants were enrolled in the study after being deemed healthy based on these results, with the exclusion criteria of a BMI below 18.5 or above 30 kg·m^−2^; high blood pressure (>140/90 mmHg); underlying health conditions which might affect metabolic function; known allergies or intolerance to drink ingredients; or regular smokers.

Fifteen young, healthy participants (age, 21 ± 1 years; BMI, 24 ± 1 kg·m^2^; male:female, 8:7) were initially recruited and consented to take part in the present study. Three participants withdrew from the study due to adverse effects (vomiting) following the ingestion of a microalgae drink, one participant became uncontactable after their first visit and therefore did not complete all conditions, and one further participant completed the study but due to an incomplete data set were not used for analysis. Therefore, excluding the above mentioned, 10 young, healthy participants (age, 21 ± 1 years; BMI, 25 ± 1 kg·m^2^; male:female, 7:3) completed this study and comprise the dataset. Participants’ characteristics (*n* = 10) are presented in Table 1.

**Table 1:** Participant characteristics and their habitual dietary intake.

Characteristic	Mean ± SEM (*n* = 10)
Sex (m:f)	7:3
Age (years)	21.3 ± 0.9
Body mass (kg)	71.6 ± 3.3
Height (m)	1.7 ± 0.0
BMI (kg·m^2^)	24.5 ± 1.2
Lean mass (kg)	56.2 ± 3.4
Body fat (%)	20.5 ± 2.9
Systolic blood pressure (mmHg)	117.6 ± 3.1
Diastolic blood pressure (mmHg)	64.7 ± 1.9
Energy intake (MJ·d^−1^)	9.3 ± 0.8
Carbohydrate intake (g·d^−1^)	247.1 ± 22.5
Carbohydrate intake (g·kg BW^−1^·d^−1^)	3.5 ± 0.3
Carbohydrate intake (En %)	44.5 ± 1.3
Protein intake (g·d^−1^)	131.2 ± 16.8
Protein intake (g·kg BW^−1^·d^−1^)	1.9 ± 0.2
Protein intake (En %)	23.5 ± 2.5
Fat intake (g·d^−1^)	81.1 ± 10.4
Fat intake (g·kg BW^−1^·d^−1^)	1.1 ± 0.1
Fat intake (En %)	31.8 ± 2.3

BMI, body mass index, BW, body weight, En, energy.

Participants were monitored by a researcher throughout each of their study test days for signs of adverse events. If a participant had an adverse event, the test day was immediately ended to manage participant comfort and avoid the adverse event influencing data collected during the postprandial period. Where an adverse event occurred and the participant had not completed all other test conditions, the entire data set was excluded (i.e., the three participants who withdrew). Adverse events (vomiting) occurred in an additional four participants who were included in the analysis. The onset of these adverse events occurred after >210 min of the test day had been completed, missing data analysis was applied for the remaining data points, accounting for <4% of data sets. For three of these participants the adverse event was during their final visit, and for one participant it occurred on their third visit, however, they were able to return for the final condition. A flowchart of the described timings of adverse events for all participants (including those withdrawn) are detailed in Supplementary material 1. Overall, two adverse events occurred following SPR ingestion, five following WCC, and one following SCC.

### 2.2 Study design

In a randomised, double-blind, crossover and counterbalanced (for order) design, participants completed four experimental test days. Randomisation was performed by an independent researcher using a computerised randomiser. During each visit, participants ingested a beverage containing 20 g protein derived from milk (MLK), spirulina (SPR), or chlorella in a whole cell (WCC) or split cell (SCC) form. Arterialised-venous blood samples were collected in the postabsorptive state and at regular time intervals over a 5 h postprandial period to assess circulating amino acid, glucose, insulin, and uric acid concentrations. Indirect calorimetry and visual analogue scales (VAS) were used at regular intervals to determine energy expenditure, and subjective appetite and palatability scores, respectively. Test days for a given participant were separated by at least 7 days to allow for complete digestion, absorption, and metabolism of the test proteins, and return to habitual dietary habits.

### 2.3 Participant diet and physical activity

Prior to taking part in the trial, participants completed a 3-day food record to assess habitual dietary intake on two weekdays and one weekend day. Records were analysed for energy and macronutrient intake using dedicated software (Nutritics Ltd.). Data are presented in Table 1. Participants were instructed to refrain from vigorous physical activity and alcohol consumption for 24 h before each test day. The evening before each test day, participants were provided with a standardised meal. Participants were allowed to choose between 2 standardised meals (energy, kJ/ carbohydrate, g/ fat, g/ protein, g: Meal 1: 1916/49/20/19, Meal 2: 2235/64/17/27) and had to adhere to the same choice prior to each experimental test day.

### 2.4 Experimental protocol

**Figure 1:**
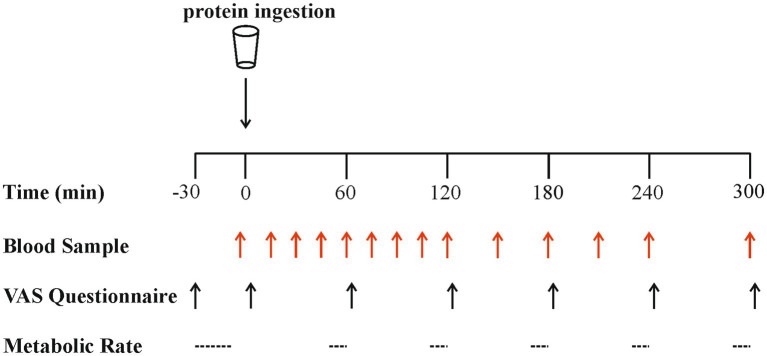
Schematic representation of the experimental protocol. VAS, visual analogue scale.

An overview of the experimental protocol is shown in Figure 1. On all test days, participants reported to the laboratory at 08:00 h after a > 12 h overnight fast and were asked to rest semi-supine on a hospital bed. A Teflon cannula was inserted retrograde into a dorsal hand vein, attached to a 0.9% saline infusion for patency (infusion rate 20 mL·h^−1^), and placed in a heated hand unit (55°C) for subsequent arterialised-venous blood sampling (47). Participants completed a visual analogue scale (VAS) to assess subjective feelings of appetite. These 100 mm paper-based scales included questions regarding hunger, satisfaction, fullness and prospective food consumption (48). Afterwards, indirect calorimetry (Cortex Metalyzer 2R gas analyser; Cortex) measurements were collected for 20 min to record resting metabolic rate. A postabsorptive blood sample was then collected (*t* = 0). Participants consumed one of the four protein beverages in a randomised and counterbalanced (for order) manner. They were instructed to consume the beverages within 5 min, and drink completion indicated the start of the postprandial period. Consumption of the beverage was followed by a 5 h postprandial period during which 11 further arterialised venous blood samples were collected at *t* = 15, 30, 45, 60, 90, 120, 150, 180, 210, 240 and 300 min, while subjects remained in a semi-supine position throughout. Further appetite VAS recordings were collected at *t* = 5, 60, 120, 180, 240 and 300 min, with additional beverage palatability questions assessing taste, aftertaste, texture and overall palatability at *t* = 5 min. The same researcher analysed VAS scales each time to minimise discrepancies, with collected data used to calculate individual and composite appetite and palatability scores as previously reported (25, 49) (see section “Calculations”). Resting metabolic rates were determined hourly at *t* = 45, 105, 165, 225, 285 for 15-min intervals. Analysis of the last 5 min of each period was used to obtain average *V̇ O_2_* and *V̇ CO_2_* values to determine energy expenditure following test drink ingestion using the modified Weir equation (50) (see section “Calculations”).

### 2.5 Experimental beverage preparation

Spirulina; SPR (Bulk™, United Kingdom), whole cell chlorella; WCC (eChlorial®, France), and split cell chlorella; SCC (Golden Greens Organic Ltd., United Kingdom) were purchased in dried powder form from commercial suppliers. Specific growing conditions of the biomasses were not provided by the suppliers. Obtainable pertinent information was as follows: WCC was cultivated in glass tubes and the cell walls kept intact (43), whereas SCC was grown in pools and after drying their cell walls broken (44). The dried microalgae powders were used as received by commercial suppliers with no additional processing performed before beverage preparation (see below). Thorough validation of cell wall disruption was not possible; however, a qualitative optical microscopy approach was taken and provided below (see “Qualitative microscopy”). All protein sources were independently analysed (Premier Analytical Services, United Kingdom) for energy, macronutrient and amino acid compositions, with the details presented in Table 2. Protein content was calculated as nitrogen (N) × 6.25 (N determined via the Kjehdal method by Premier Analytical Services, United Kingdom). While the presence of non-protein nitrogen-containing factors potentially introduces a small amount of error, we consider this to be in line with what is typically accepted within the food industry (51) and similar studies within the literature (24, 25, 52). To ensure isonitrogenous and isovolumetric conditions could be achieved across beverages, the milk protein beverage consisted of commercially obtained instant full cream milk powder (Nestlé UK Ltd., United Kingdom) dissolved in skimmed milk (Tesco Stores Ltd., United Kingdom).

**Table 2:** Nutritional content of the prepared test drinks.

	MLK	SPR	SCC	WCC
Macronutrient content				
Energy (kJ)	1991.0	1681.8	1739.3	1770.4
Energy (kcal)	471.3	396.4	410.3	417.9
Protein (g)	20.0	20.0	20.0	20.0
Carbohydrate (g)	75.0	75.0	75.0	75.0
Fat (g)	10.1	0.8	2.1	2.6
Fibre (g)	0.0	2.9	4.5	5.8
Amino acid content (g)				
Alanine	0.7	1.6	1.4	1.3
Arginine	0.7	1.4	1.0	1.0
Aspartic Acid	1.8	2.1	1.5	1.6
Cysteine	–	–	0.2	0.2
Glutamic Acid	4.7	2.8	2.0	1.8
Glycine	0.4	1.0	0.9	0.9
Histidine	0.6	0.3	0.3	0.4
Isoleucine	1.1	1.1	0.4	0.5
Leucine	2.2	1.8	1.2	1.2
Lysine	1.9	1.0	1.3	1.6
Methionine	–	–	0.4	0.4
Phenylalanine	1.1	0.9	0.7	0.7
Proline	2.0	0.7	0.8	0.8
Serine	1.3	1.0	0.7	0.7
Threonine	1.0	1.1	0.7	0.8
Tryptophan	–	–	–	–
Tyrosine	1.0	0.9	0.6	0.6
Valine	1.4	1.3	0.7	0.7
EAA	9.2	7.6	5.8	6.2
NEAA	12.7	11.5	9.1	9.0
TAA	21.8	19.1	14.9	15.2

MLK, milk; SPR, spirulina; SCC, split cell chlorella; WCC, whole cell chlorella; EAA, essential amino acids; NEAA, non-essential amino acids; TAA, sum of measured amino acids.

Protein beverages were prepared the evening before test days by adding the amount of powder required (43, 32, 36, and 34 g for MLK, SPR, WCC and SCC, respectively) to provide 20 g protein to 200 mL water, or 200 mL skimmed milk (MLK condition). Maltodextrin (Myprotein, United Kingdom) was added to make each drink up to a total of 75 g of carbohydrates. Fifteen mL of artificial energy-free vanilla flavouring (Jordan’s Skinny Mixes, UK) and 10 mL of green energy-free food colouring (Tesco Stores Ltd., United Kingdom) were added for blinding purposes (making all drinks a dark green colour) and mixed for approximately 2 min using a food blender. Following drink consumption by the participant, an additional 100 mL was added to ‘rinse’ the bottle and ensure that all protein had been consumed, making a total fluid volume of 400 mL consumed by participants on each occasion. Double-blinding of the drinks was achieved by having a different researcher to the one performing the experiment prepare the drinks in a metal, non-transparent bottle ready for consumption. Drinks were refrigerated at 4°C until use. Following ingestion of each drink, volunteers were asked to identify which condition they thought they had received which was noted down without feedback. The overall success rate for volunteers correctly identifying the condition was 60%. Individual successful identification rates were as follows: SPR, 40%; MLK, 100%; SCC, 40%; WCC, 60%, implying partially successful blinding, with the exception of MLK.

### 2.6 Blood sampling and subsequent analysis

Eight mL of arterialised venous blood were collected into a syringe at each sampling point. For each blood sample a 20 μL plastic capillary was filled and immediately analysed for blood glucose concentrations (Biosen C-Line GP+, EKF diagnostics). The remaining whole blood was split equally into serum separator tubes (BD Vacutainer SST II tubes, BD Diagnostics), and lithium heparin-containing tubes (BD Vacutainer LH; BD Diagnostics). The blood tubes were handled according to the manufacturer’s instructions (serum separator tubes were left at room temperature for 30 min to clot prior to centrifugation, lithium-heparin tubes were centrifuged immediately). Following centrifugation (1,300 ×g at 4°C for 10 min) the resulting blood serum and blood plasma supernatants were aliquoted and stored at −80°C until subsequent analysis.

Serum uric acid concentrations were measured enzymatically via colorimetry on a Cobas 8,000 automated analyser (Roche Diagnostics) as described previously (37). Serum insulin concentrations were determined using a commercially available ELISA assay kit (DRG Insulin ELISA, EIA-2935, DRG International Inc., United States) following the manufacturer’s instructions.

Plasma concentrations of alanine, glutamic acid, glycine, histidine, isoleucine, leucine, lysine, methionine, phenylalanine, proline, serine, threonine, tyrosine, and valine were determined by GC–MS with electron impact ionisation (Agilent) as previously described (25, 52). Briefly, to prepare samples for GC–MS, 10 μL of 2 mM norleucine were added as an internal standard to 500 μL of plasma and deproteinised on ice with 500 μL of 15% ^w^/_v_ 5-sulfosalcylic acid. Samples were then vortexed and centrifuged at 4000 ×g for 10 min at 4°C. The supernatant was then loaded onto cation-exchange columns. Columns were filled with ddH_2_O, followed by 6 mL 0.5 M acetic acid and then washed 5 more times with ddH_2_O. Amino acids were then eluted from the columns with 2 mL of 6 M ammonium hydroxide. The eluate was dried using a Speed-Vac at 60°C and then derivatised via the addition of 50 μL of N-tert-butyldimethylsilyl-N-methyltrifluoroacetamide1% tertbutyl-dimethylchlorosilane and 50 μL of acetonitrile, followed by heating at 95°C for 45 min. Derivatized samples were subsequently analysed on a GC–MS as previously described (25, 52).

### 2.7 Qualitative microscopy

Optical brightfield microscopy was used to qualitatively observe any differences in the commercial suppliers described whole cell- and split cell-chlorella preparations. See Supplementary material 2 for details.

### 2.8 Calculations

Modified Weir equation (50):


$$\begin{array}{l} \mathrm{Resting}\;\mathrm{energy}\;\mathrm{expenditure}\;\left(\mathrm{kcal}.24\;h^{-1}\right)\\ \;=\left(\left(3.941\times VO_{2}\right)+\left(1.106\times VCO_{2}\right)\right)\times 1440 \end{array}$$

Appetite score (49):


$$\mathrm{Appetite}\;\mathrm{score}=\frac{\left(\begin{array}{l} \mathrm{hunger}+\left(100-\mathrm{fullness}\right)+\mathrm{desire}\;to\;\mathrm{eat}\\ \;+\mathrm{prospective}\;\mathrm{food}\;\mathrm{consumption} \end{array}\right)}{4}$$

### 2.9 Statistical analysis

Based on previous research using a similar randomised cross-over design (53, 54), sample size was calculated with differences in postprandial plasma essential amino acid (EAA) incremental area under the curve (iAUC) between protein sources as the primary outcome measure. A sample size of 12 participants was calculated with a power of 80% and a significance level of 0.05 to detect a relevant difference in EAA iAUC between protein sources.

For each parameter, all four conditions were compared within the same statistical test and analysed by a two-way ANOVA with repeated measures (with condition and time as factors). In the event of a significant main effect, Tukey’s multiple comparisons tests were applied to locate individual differences. Or, where the iAUC was calculated, a one-way ANOVA was performed to detect significant effects of condition. If a significant main effect was detected, Tukey’s multiple comparisons tests were again applied to locate individual differences. Statistical significance was set at *p* < 0.05. *N* = 10 unless stated otherwise, with missing data handled using expected-maximisation algorithm. All statistical analysis were performed by using GraphPad Prism version 10 (GraphPad Software). All data are expressed as means with their standard errors.

## 3 Results

### 3.1 Plasma amino acid concentrations

**Figure 2:**
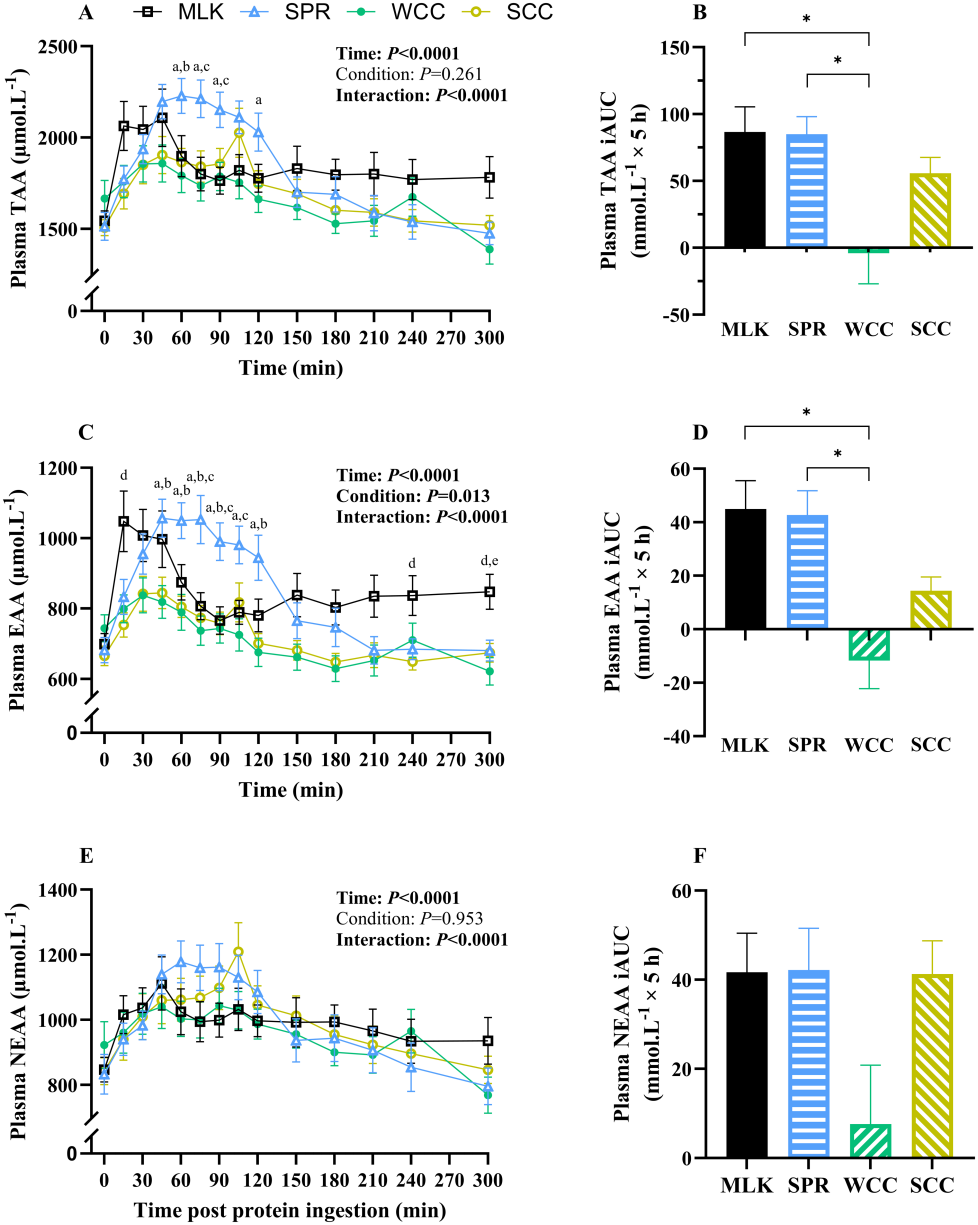
Plasma total **(A)**, total essential **(C)** and total non-essential **(E)** amino acid concentrations in the fasting state (*t* = 0) and at regular intervals during a 5-h postprandial period following the ingestion of 20 g milk protein (**□**, MLK), spirulina protein (***Δ***, SPR), whole cell chlorella protein (●, WCC), or split cell chlorella protein (**○**, SCC) in healthy young adults (*n* = 10). Values are means, with their standard errors represented by vertical bars. Time course data were statistically analysed with a two-way repeated measures ANOVA, significant *p* values are written in bold font. Tukey’s multiple comparisons test was applied where appropriate to locate individual differences: a, b, c, d and e indicate values significantly different (*p* < 0.05) between SPR vs. WCC, SPR vs. SCC, SPR vs. MLK, MLK vs. SCC, and MLK vs. WCC, respectively. Data are also expressed as iAUC for the total 5-h postprandial responses for total **(B)**, total essential **(D)** and total non-essential **(F)** amino acids. These data were analysed for a main effect with a one-way ANOVA, and Tukey’s multiple comparisons test applied to locate individual differences: * indicate values significantly different (*p* < 0.05) from each other for MLK, SPR, WCC and SCC conditions.

Postabsorptive (i.e., *t* = 0) and postprandial plasma total amino acid (TAA), essential amino acid (EAA), and non-essential amino acid (NEAA) concentrations following protein drink ingestion are shown in Figures 2A,C,E. From similar postabsorptive levels, plasma TAA, EAA and NEAA concentrations all increased following drink ingestion (time effects; all *p* < 0.001), although to different extents between groups (time x condition interactions; all *p* < 0.001).

Postabsorptive plasma TAA concentrations (Figure 2A) for MLK, SPR, WCC and SCC were 1,544 ± 105, 1,514 ± 127, 1,666 ± 144 and 1,507 ± 109 μmol·L^−1^, respectively (*p* = 0.38). Post drink ingestion, plasma TAA concentrations reached peaks of 2,361 ± 121, 2,344 ± 88, 2002 ± 99 and 2093 ± 126 μmol·L^−1^ after 72 ± 26, 78 ± 8, 57 ± 13 and 81 ± 7 min, in MLK, SPR, WCC and SCC, respectively. For SCC there was a ‘double peak’ around 45 min and again at 105 min. These data translated to different postprandial TAA availabilities between conditions, reflected by postprandial iAUC (above the baseline value) varying significantly (Figure 2B, *p* = 0.002) depending on protein source (MLK; 86.6 ± 17.8, SPR; 84.9 ± 12.5, WCC; −4.1 ± 21.7, SCC; 55.7 ± 11.3 mmol·L^−1^). Specifically, TAA iAUC following SPR and MLK ingestion were similar (*p* = 0.99), but both were higher than WCC (*p* < 0.05) but not SCC (*p* > 0.05). SCC showed a trend for higher TAA iAUC compared with WCC (*p* = 0.09).

A similar pattern was seen with plasma EAA (Figure 2C), where from similar postabsorptive plasma concentrations (MLK 698 ± 41; SPR 681 ± 48; WCC 744 ± 54; SCC 665 ± 42 μmol·L^−1^, *p* = 0.39), peak EAA concentrations differed (*p* < 0.05) between MLK and SPR (1,171 ± 64 and 1,139 ± 52 μmol·L^−1^, respectively) vs. WCC and SCC (881 ± 48 and 894 ± 48 μmol·L^−1^, respectively). This translated to different postprandial EAA availabilities, reflected by iAUC varying significantly (Figure 2D, *p* = 0.0002) depending on protein source (MLK; 45.0 ± 10.0, SPR; 42.7 ± 8.6, WCC; −11.8 ± 9.9, SCC; 14.3 ± 4.9 mmol·L^−1^). Specifically, EAA iAUC following SPR and MLK ingestion were similar (*p* = 0.99), both were higher than WCC (*p* < 0.001), but not SCC (*p* > 0.05). No differences were detected between chlorella conditions (*p* = 0.19).

For the NEAA (Figure 2E), postabsorptive plasma concentrations were similar across conditions (MLK 846 ± 63; SPR 832 ± 79; WCC 922 ± 90; SCC 842 ± 66 μmol·L^−1^, *p* = 0.64) and the peak NEAA concentrations did not differ (1,221 ± 73, 1,250 ± 59, 1,156 ± 57 and 1,224 ± 87 μmol·L^−1^ for MLK, SPR, WCC and SCC, respectively, *p* > 0.05). The NEAA postprandial iAUC (Figure 2F) varied depending on protein source (MLK; 41, 0.7 ± 8.6, SPR; 42.1 ± 8.9, WCC; 7.6 ± 12.5, SCC; 41.3 ± 7.1 mmol·L^−1^, *p* = 0.046); however, multiple comparisons did not detect individual differences between drinks.

The iAUC for WCC was negative when calculated for the entire 5 h postprandial period for TAA and EAA (not NEAA). However, when the postprandial period was split into 0–2.5 h and 2.5–5 h periods the WCC iAUC was positive for the first half of the postprandial period. For 0–2.5 h significant differences were detected (*p* > 0.05) for TAA between WCC compared with all other conditions (MLK, SPR and SCC). This is illustrated in Supplementary material 3.

Data showing the postprandial plasma responses of each measured individual amino acid are displayed in Supplementary material 4. All individual amino acid plasma concentrations reported a time effect (*p* < 0.05), except glutamic acid (*p* = 0.19). Differences between conditions (*p* < 0.05) were only detected for isoleucine, leucine, methionine and tyrosine. Time × condition interactions were reported for all the measured individual amino acid plasma concentrations, except histidine (*p* = 0.25) and lysine (*p* = 0.13).

### 3.2 Blood glucose and serum insulin concentrations

**Figure 3:**
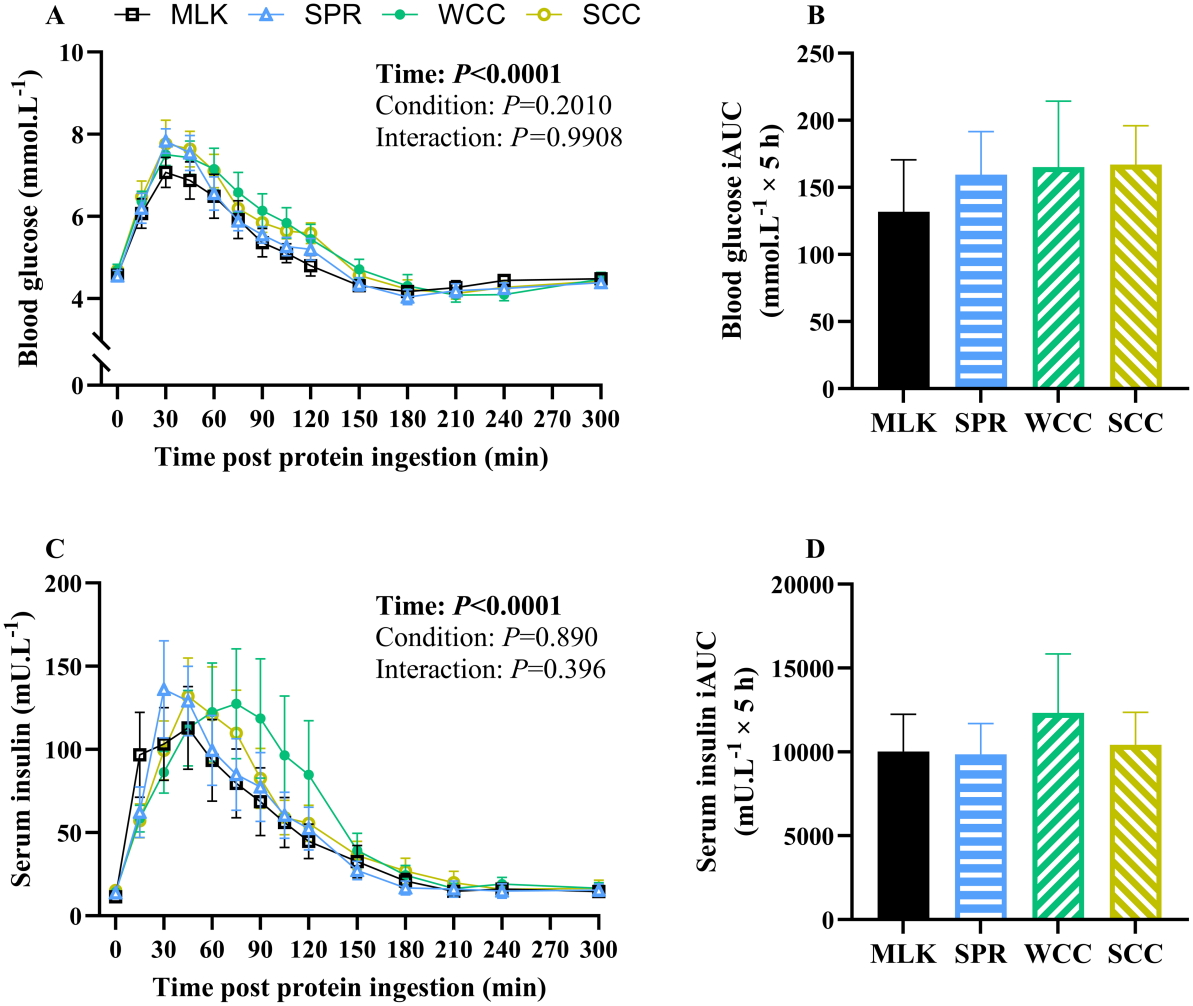
Whole blood glucose **(A)** and serum insulin **(C)** concentration in the fasting state (*t* = 0) and at regular intervals during a 5-h postprandial period following the ingestion of 20 g milk protein (**□**, MLK), spirulina protein (**Δ**, SPR), whole cell chlorella protein (●, WCC), or split cell chlorella protein (**○**, SCC) in healthy young adults (*n* = 10). Data are also expressed as incremental area under the curve (iAUC) for the total 5 h postprandial response for whole blood glucose **(B)** and serum insulin **(D)**. Values are means, with their standard errors represented by vertical bars. Time course data were analysed with a two-way repeated measures ANOVA, iAUC data were analysed for a main effect with a one-way ANOVA. Significant *p* values (*p* < 0.05) are written in bold font.

From similar postabsorptive values, blood glucose concentrations (Figure 3A) increased after drink ingestion (time effect; *p* < 0.001) but with no differences between conditions (condition and time × condition interaction; *p* = 0.20 and 0.99, respectively). Postprandial blood glucose iAUC did not differ between conditions (Figure 3B, *p* = 0.91).

Postabsorptive and postprandial serum insulin concentrations are presented in Figure 3C. From similar postabsorptive concentrations (MLK, 11 ± 2; SPR, 14 ± 2; WCC, 14 ± 3; SCC, 15 ± 2 mU·L^−1^; *p* = 0.74), serum insulin concentrations increased following drink ingestion (time effect; *p* < 0.001), but with no differences between conditions (condition and time × condition interaction; *p* = 0.89 and 0.40, respectively). Postprandial insulin iAUC also did not differ between conditions (Figure 3D, *p* = 0.89).

### 3.3 Whole body energy expenditure

**Figure 4:**
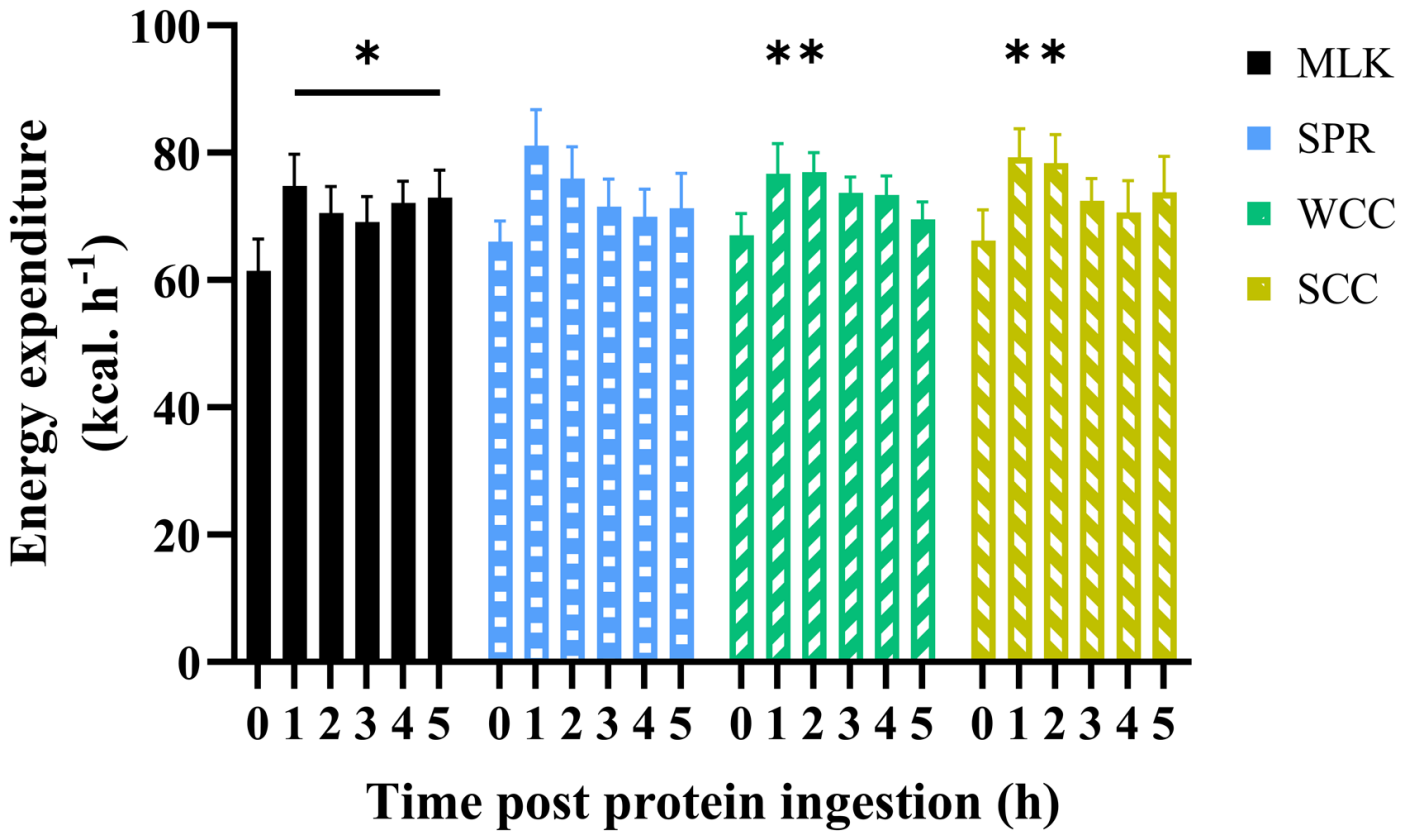
Energy expenditure in the fasting (*t* = 0) state and at hourly intervals during the 5 h postprandial period following the ingestion of 20 g milk protein (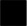
MLK), spirulina protein (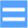
SPR), whole cell chlorella protein (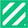
WCC), or split cell chlorella protein (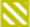
SCC) in healthy young adults (*n* = 9). Values are means, with their standard errors represented by vertical bars. Data were analysed with a two-way repeated measures ANOVA and Tukey’s multiple comparisons test to locate individual differences: * indicates values significantly different from their corresponding fasting value (*p* < 0.05).

Resting whole-body energy expenditure in the postabsorptive state and at regular intervals during the 5 h postprandial period is displayed in Figure 4. Postabsorptive resting energy expenditure was equivalent between conditions (MLK, 61 ± 5 [1,475 ± 113]; SPR 66 ± 3 [1,585 ± 74]; WCC, 67 ± 3 [1,609 ± 77]; SCC, 66 ± 5 [1,588 ± 110] kcal· h^−1^ [kcal·24 h^−1^]; *p* = 0.78). Drink ingestion resulted in a significant increase in energy expenditure from postabsorptive values (time effect; *p* < 0.001), and for the entire duration of the postprandial period for MLK (*p* < 0.05), and for 2 h following the ingestion of WCC and SCC (*p* < 0.05). However, no differences between conditions were detected (condition and time × condition interaction; *p* = 0.94 and 0.10, respectively).

### 3.4 VAS responses

**Figure 5:**
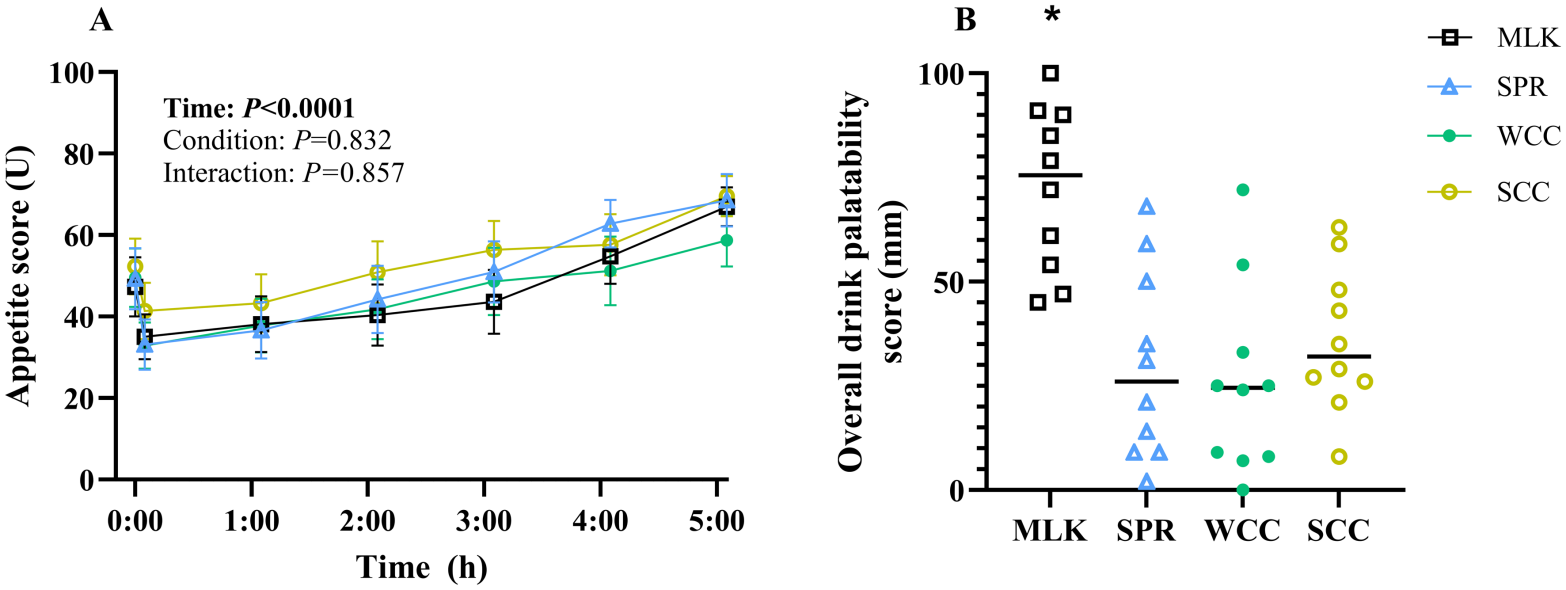
**(A)** Participant subjective appetite scores measured by Visual Analogue Scale in the fasting state (*t* = 0) and at regular intervals during a 5 h postprandial period following the ingestion of 20 g milk protein (**□**, MLK), spirulina protein (**Δ**, SPR), whole cell chlorella protein (●, WCC), or split cell chlorella protein (**○**, SCC) in healthy young adults (*n* = 10). Values are means, with their standard errors represented by vertical bars. For this time course data, all conditions were statistically analysed with a two-way repeated measures ANOVA. Significant *p* values (*p* < 0.05) are written in bold font. **(B)** Overall drink palatability scores of participants were recorded by Visual Analogue Scale, 5 min following the ingestion of 20 g milk protein (**□**, MLK), spirulina protein (**Δ**, SPR), whole cell chlorella protein (●, WCC), or split cell chlorella protein (**○**, SCC) in healthy young adults (*n* = 10). All conditions were statistically analysed with a one-way ANOVA (*p* < 0.0001) and Tukey’s multiple comparison test applied to locate individual differences. * indicates significant difference (*p* < 0.05).

Participants’ subjective composite appetite scores during the experimental trials are presented in Figure 5A. Individual ratings of hunger, fullness, prospective food intake, and desire to eat are presented in Supplementary material 5. Following drink ingestion, main effects of time for all appetite variables were observed (all *p* < 0.001), but no condition effect (*p* = 0.83) or time x condition effects (*p* = 0.90).

A subjective ‘overall palatability’ score for each condition is presented in Figure 5B, with values recorded of MLK 72 ± 6 mm, SPR 30 ± 7 mm, WCC 26 ± 7 mm and SCC 36 ± 5 mm. Palatability scores for taste, smell, aftertaste, and texture are presented in Supplementary material 6. Scores of each palatability variable, including overall palatability, differed between protein sources (condition effect; all *p* < 0.05). Across all variables, except texture, MLK scored higher than all microalgae drinks (*p* < 0.02), and there were no differences between the microalgae drinks (*p* > 0.05). For texture, the only difference detected was MLK scoring higher than WCC (*p* < 0.03).

### 3.5 Serum uric acid concentration

**Figure 6:**
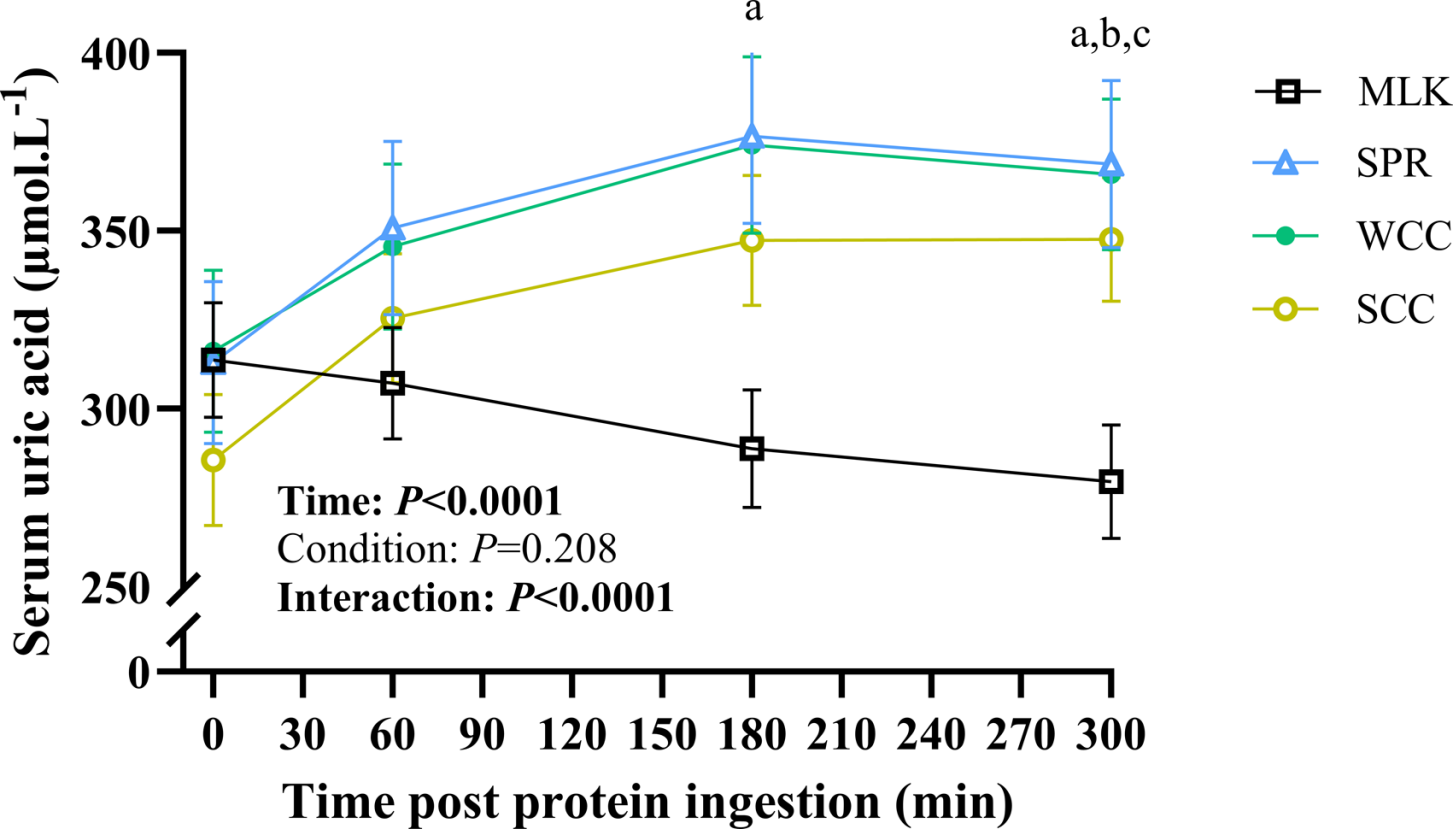
Serum uric acid concentration in the fasting state (*t* = 0) and at intervals (60, 180 and 300 min) during the 5-h postprandial period following the ingestion of 20 g milk protein (**□**, MLK), spirulina protein (**Δ**, SPR), whole cell chlorella protein (●, WCC), or split cell chlorella protein (**○**, SCC) in healthy young adults (*n* = 10). Values are means, with their standard errors represented by vertical bars. All conditions were statistically analysed with a two-way repeated measures ANOVA and Tukey’s multiple comparison test applied to locate individual differences (*p* < 0.05): a, b and c indicate values significantly different between SPR vs. MLK, MLK vs. SCC, and MLK vs. WCC, respectively.

Serum uric acid concentrations in the postabsorptive state and over the 5 h postprandial period are shown in Figure 6. Postabsorptive serum uric acid concentrations were similar in all conditions (MLK, 314 ± 15; SPR, 313 ± 22; WCC, 316 ± 22; SCC, 286 ± 17; *p* = 0.68). There was a significant time (*p* < 0.0001) and time × condition interaction (*p* < 0.0001), but no significant effect of the condition alone (*p* = 0.21). In the MLK condition, serum uric acid concentrations decreased steadily across the postprandial period, whereas for the microalgae conditions (SPR, WCC and SCC) serum uric acid concentrations increased by 1 h and remained elevated for the duration of the postprandial period, such that at 300 min post drink ingestion, differences were detected between MLK and all microalgae conditions (*p* < 0.05; with no individual differences postprandially between microalgae conditions).

### 3.6 Qualitative cell disruption

See Supplementary material 2 for optical brightfield microscopy images of whole cell- and split cell-chlorella. Observationally, fewer cell clusters were observed in the split cell compared with the whole cell-chlorella preparation.

## 4 Discussion

In the present study, we assessed the postprandial metabolic responses of 10 healthy young adults to the ingestion of isonitrogenous (20 g protein) (55, 56) and carbohydrate matched (75 g) (46) boluses of the cyanobacteria, spirulina, and the microalgae, chlorella (in a whole cell and split cell preparation), in comparison with milk protein as a commonly studied reference protein (57, 58). We first confirmed that commercially available spirulina, whole cell- and split cell-chlorella were rich in protein (62, 56 and 58% of total dry mass, respectively) and essential amino acids (EAA; 40, 41 and 39% of total protein, respectively). We then show broadly similar postprandial metabolic responses (e.g., diet induced thermogenesis, plasma glucose clearance, satiety etc.) following ingestion of all these protein sources, with the exception of postprandial circulating amino acid and uric acid concentrations. Specifically, total postprandial amino acid availability was greater with milk and spirulina compared with chlorella, though split cell chlorella tended to be higher than whole cell chlorella. Finally, irrespective of microalgae/cyanobacteria species, their ingestion raised serum uric acid concentrations to near clinically significant levels, while milk did not.

Postprandial metabolism is affected by dietary protein ingestion beyond just the provision of amino acids as precursors for protein synthesis, by contributing towards insulin secretion, glycaemic control, dietary induced thermogenesis and appetite regulation (33–36, 59). Establishing these responses to microalgae ingestion is paramount before progressing them as a viable protein source. Hence, to examine any potential role of differing amino acid compositions, macro- and micro-nutrient contents, and food matrices between the diverse food sources on glycaemic control, we added simple carbohydrate to the drinks to a total of 75 g, a strategy that mimics a mixed meal tolerance test (33, 45, 46). Though the potent insulin response to simple carbohydrates likely masked our ability to detect differences in the insulinotropic potential of the different protein sources, it did allow us to show that the postprandial glucose responses did not differ between conditions, indicating similar levels of glycaemic control. Furthermore, we report that the microalgae conditions appropriately induced a dietary thermogenic effect, and satiety response, to the same extent as milk protein. It is therefore reasonable to assume that microalgae, as a bulk source of protein and under mixed meal conditions, are able to regulate postprandial metabolism in the same way as more traditional proteins (i.e., milk).

We then assessed microalgae protein in terms of its ability to deliver amino acids to the circulation (i.e., bioavailability). In line with our assumption of milk as an appropriate high quality animal protein to use as a control condition, the milk protein drink used in the present work was EAA rich (42%), and its ingestion resulted in rapid increases in blood concentrations of all amino acids, followed by a gradual decline across the postprandial period. This response is in line with previous work (15, 42, 58, 60) and is indicative of a high quality and bioavailable dietary protein source (42, 61). Spirulina ingestion elicited similar peak plasma total amino acid responses (both in speed [milk; 72, spirulina; 78 min] and magnitude [milk; 1,544, spirulina; 1,514 μmol·L^−1^]), and plasma availability of total amino acids, EAAs and non-EAAs across the 5 h postprandial period (indicated by iAUC). These data are in line with recent work (21, 24, 25), and confirmed our hypothesis that spirulina represents a viable protein source that appears to be effectively digested and absorbed and, as a result, implies a similar amino acid bioavailability to milk protein. The present literature typically describes plant derived proteins as less bioavailable than animal proteins, which limits their ability to stimulate muscle protein synthesis (18) and the adaptive response to exercise training (19, 62). Therefore, our data of a more amino acid balanced, non-animal protein source, and achieving equivalent amino acid bioavailability to milk (a high-quality animal derived protein source) shows promise for microalgae to occupy a role in supporting a sustainable food future.

Chlorella ingestion, on the other hand, resulted in lower total postprandial plasma amino acid availabilities compared with milk and spirulina, albeit with small improvements noted in split vs. whole cell preparations. Although whole cell chlorella ingestion resulted in the quickest postprandial plasma amino acid peak (57 min) of all the protein sources, it also exhibited the smallest magnitude peak (2002 μmol·L^−1^) and had the lowest 5 h total amino acid availability. In fact, we reported an overall negative total plasma amino acid and EAA availability for whole cell chlorella across the 5 h postprandial period, driven by negative values from 2.5–5 h after protein ingestion compared to baseline (but not at 0–2.5 h). This suggests that postprandial plasma amino acid disappearance (i.e., protein synthesis and oxidation, stimulated as a result of rises in circulating amino acids and insulin) was greater than the exogenous appearance from the chlorella (15, 16), underlining its poor digestibility.

This interpretation extends to most (grouped) amino acids we observed; for instance, the differences in EAA content between milk and split cell chlorella, and milk and whole cell chlorella were 37 and 33% respectively, yet the differences in plasma EAA 5 h iAUC were 68 and 126%, respectively. Similarly, the difference in leucine contents between milk and split cell chlorella or whole cell chlorella were 45%, yet 64 and 153% differences existed in its postprandial availability, respectively. These data are broadly in line with our previous work (24, 25) though here seemingly less (statistically at least) striking. This reduced effect size may be explained by the present work serving a smaller protein dose minimising differences between species, including the co-ingestion of carbohydrate likely slowing digestion and/or absorption allowing for greater intestinal microvilli transit exposure (63), or using different commercial suppliers and therefore different algal cultivation and processing. Collectively, the present work paired with our previous data (24, 25) indicate that, despite chlorella being a protein and essential amino acid rich potential food source, it is considerably less bioavailable than a range of other protein sources including plant proteins such as pea and lupin. Though we have previously reported that this does not appear to limit its potential to acutely stimulate muscle protein synthesis rates (24), it does imply chlorella may be sub-optimal as a future protein source applied more broadly within human nutrition.

Notably, our ability to observe small *in vivo* differences in postprandial amino acid availability of different chlorella preparations, with commercially reported ‘intact cells’ (43) or ‘broken cell walls’ (44) is encouraging that further optimisation is possible. For instance, in Supplementary Figure 2 we detail our qualitative efforts to explore the effectiveness of the reported cell disruption with basic microscopy; fewer cell clusters were observed in split cell chlorella compared with whole cell chlorella - cell clusters in both preparations are likely formed during (spray) drying (64). This suggests some disruption to the crude biomass, but visually most cells still appear intact. Since chlorella possesses a complex multi-layered cell wall consisting of various insoluble and indigestible polysaccharides (10, 26), more vigorous cell disruption techniques may increase the fragmenting of cell walls, thereby improving chlorella digestibility. This is supported by experiments performed *in vitro* and in animal models, where optimising cell disruption methods improve indirect measures (e.g., protein yields, faecal protein calculations etc.) of digestibility (26–28, 30). Such processing appears unnecessary for spirulina as it is evidently more digestible likely due to its less robust gram-negative cell wall type.

In addition to cell wall structures, the divergent postprandial amino acid responses following chlorella or spirulina ingestion may also be influenced by other species differences, such as differing inherent protein types. For example, spirulina contains the light harvesting pigment-protein complex phycocyanin, which does not occur in green algae (e.g., chlorella). Phycocyanin accounts for ~20% of spirulina protein, is soluble in water and, importantly, is rapidly digested by pepsin (65), making human digestion feasible. Contrastingly, 20% of chlorella proteins (including light harvesting membrane proteins) are bound to the cell wall (27, 66), which may be speculated to reduce bioaccessibility to the amino acids. Finally, in these preparations, spirulina had roughly half the fat and fibre content compared to both chlorella preparations (Table 2). Higher content of these macronutrients are generally reported to delay protein digestibility (67–69), possibly contributing to differing postprandial amino acid concentrations. Future food science approaches designed to exploit microalgae as a bulk protein source for human nutrition should therefore consider these inherent species differences.

Aside from challenges associated with improving microalgae (specifically chlorella) protein bioavailability, our data raised broader issues concerning the suitability for human consumption, previously unaddressed due to only small quantities being ordinarily consumed. For example, the 20 g serving of microalgae protein increased concentrations of serum uric acid to near clinically significant levels (i.e., >420 and > 360 μmol·L^−1^ for men and women, respectively) (37, 70) for the entire 5 h postprandial period. Mean maximum uric acid concentrations following spirulina, split cell chlorella and whole cell chlorella ingestion reached 397, 361, and 403 μmol·L^−1^ for male participants and 332, 330, and 318 μmol·L^−1^ in female participants, respectively. Similar elevations of circulating uric acid have only been previously reported across multi-day microalgae ingestion studies (39, 71). Although it is yet to be determined if these short-term elevations directly translate to impairments in longer term health, there is an established association between circulating uric acid concentrations and various cardio-metabolic conditions (38, 39, 71). This therefore poses a potential barrier to recommendations of widespread human consumption of microalgae protein. Processing techniques have previously been applied in other foods to obviate these concerns (e.g., heat treatment reducing nucleic acid content in mycoprotein) (37), and so analogous approaches should be explored for potential algal based foods. Furthermore, we also report clear palatability issues with microalgae, both with respect to acceptability (e.g., low taste and smell scores) and frequency of adverse responses (vomiting). The apparent >2 h onset required for adverse responses to manifest may imply the co-ingestion of carbohydrate and the previously mentioned consequent delay in amino acid absorption may also alter the metabolism and transit of other noxious (micro)nutrients. The observation that no such events took place in previous work (24, 25) that did not co-ingest carbohydrate supports this interpretation. Whether this would remain a problem if microalgae were incorporated into future foods remains to be established.

## 5 Conclusion

We conclude that microalgae/cyanobacteria may represent a bioavailable source of dietary protein *in vivo* in humans, although consideration to species selection and/or biomass processing is required. Our data confirm that spirulina is comparable in its ability to provide postprandial systemic amino acids with a high-quality animal protein, and therefore its incorporation into human diets should be considered as a sustainable protein alternative. Chlorella protein however, appears less bioavailable than milk and spirulina, with some improvement apparent when consumed after the disruption of its cell wall. It is clearly desirable for future work to optimise microalgae cell wall disruption methods such that they can maximise nutritional potential, yet be carried out in a scalable and environmentally and economically sustainable manner. In addition, both spirulina and chlorella demonstrated some challenges in their palatability, tolerability, and elevation of serum uric acid concentrations post ingestion, which all represent significant hurdles for their utility in the food industry. These areas require addressing from multidisciplinary angles prior to viewing microalgae as a bulk protein source for incorporation into human diets to ensure food safety and consumer uptake.

Despite these challenges that our work has identified, this proof-of-concept data show that with further development, microalgae offer promise as a sustainable alternative protein source that can contribute to future protein requirements of human diets.

## Data Availability

The original contributions presented in the study are included in the article/Supplementary material, further inquiries can be directed to the corresponding author.

## References

[1] Jäger R Kerksick CM Campbell BI Cribb PJ Wells SD Skwiat TM. International Society of Sports Nutrition Position Stand: protein and exercise. J Int Soc Sports Nutr. (2017) 14:20. doi: 10.1186/s12970-017-0177-8, PMID: 28642676 PMC5477153

[2] Gibbs J Cappuccio FP. Plant-based dietary patterns for human and planetary health. Nutrients. (2022) 14:1–11. doi: 10.3390/nu14081614 PMC9024616 35458176

[3] Wood P Tavan M. A review of the alternative protein industry. Curr Opin Food Sci. (2022) 47:100869. doi: 10.1016/j.cofs.2022.100869

[4] Morach B Witte B Walker D von Koeller E Grosse-Holz F Rogg J. Food for thought: the protein transformation. Industrial Biotechnol. (2021) 17:125–33.

[5] Mosibo OK Ferrentino G Udenigwe CC. Microalgae proteins as sustainable ingredients in novel foods: recent developments and challenges. Food Secur. (2024) 13:733. doi: 10.3390/foods13050733, PMID: 38472846 PMC10930894

[6] Moura MAF Martins BA Takahashi JA. Alternative protein sources of plant, algal, fungal and insect origins for dietary diversification in search of nutrition and health. Crit Rev Food Sci Nutr. (2023) 63:10691–708. doi: 10.1080/10408398.2022.2085657, PMID: 35698908

[7] Diaz CJ Douglas KJ Kang K Kolarik AL Malinovski R Torres-Tiji Y. Developing algae as a sustainable food source. Front Nutr. (2023) 9:1–21. doi: 10.3389/fnut.2022.1029841 PMC9892066 36742010

[8] Williamson E Ross IL Wall BT Hankamer B. Microalgae: potential novel protein for sustainable human nutrition. Trends Plant Sci. (2023) 29:370–82. doi: 10.1016/j.tplants.2023.08.006, PMID: 37690907

[9] Wang Y Tibbetts S McGinn P. Microalgae as sources of high-quality protein for human food and protein supplements. Food Secur. (2021) 10:1–18. doi: 10.3390/foods10123002 PMC8700990 34945551

[10] Ross IL Shah S Hankamer B Amiralian N. Microalgal nanocellulose – opportunities for a circular bioeconomy. Trends Plant Sci. (2021) 26:924–39. doi: 10.1016/j.tplants.2021.05.004, PMID: 34144878

[11] Meganaharshini M Sudhakar V Dhivya Bharathi N Deepak S. Review on recent trends in the application of protein concentrates and isolates – a food industry perspective. Food Humanit. (2023) 1:308–25. doi: 10.1016/j.foohum.2023.05.022

[12] Koyande AK Chew KW Rambabu K Tao Y Chu DT Show PL. Microalgae: a potential alternative to health supplementation for humans. Food Sci Human Wellness. (2019) 8:16–24. doi: 10.1016/j.fshw.2019.03.001

[13] Bortolini DG Maciel GM Fernandes IAA Pedro AC Rubio FTV Branco IG. Functional properties of bioactive compounds from Spirulina spp.: current status and future trends. Food Chem (Oxf). (2022) 5:100134. doi: 10.1016/j.fochms.2022.100134 36177108 PMC9513730

[14] García JL de Vicente M Galán B. Microalgae, old sustainable food and fashion nutraceuticals. Microb Biotechnol. (2017) 10:1017–24. doi: 10.1111/1751-7915.12800 28809450 PMC5609256

[15] Groen BBL Horstman AM Hamer HM de Haan M van Kranenburg J Bierau J. Post-prandial protein handling: you are what you just ate. PLoS One. (2015) 10:1–22. doi: 10.1371/journal.pone.0141582 PMC4640549 26556791

[16] Mitchell WK Wilkinson DJ Phillips BE Lund JN Smith K Atherton PJ. Human skeletal muscle protein metabolism responses to amino acid nutrition. Adv Nutr. (2016) 7:828S–38S. doi: 10.3945/an.115.011650, PMID: 27422520 PMC4942869

[17] Pinckaers PJM Trommelen J Snijders T van Loon LJC. The anabolic response to plant-based protein ingestion. Sports Med. (2021) 51:59–74. doi: 10.1007/s40279-021-01540-8 34515966 PMC8566416

[18] Berrazaga I Micard V Gueugneau M Walrand S. The role of the anabolic properties of plant-versus animal-based protein sources in supporting muscle mass maintenance: a critical review. Nutrients. (2019) 11:1825. doi: 10.3390/nu11081825, PMID: 31394788 PMC6723444

[19] Lim MT Pan BJ Toh DWK Sutanto CN Kim JE. Animal protein versus plant protein in supporting lean mass and muscle strength: a systematic review and Meta-analysis of randomized controlled trials. Nutrients. (2021) 13:1–18. doi: 10.3390/nu13020661 PMC7926405 33670701

[20] Gorissen SHM Crombag JJR Senden JMG Waterval WAH Bierau J Verdijk LB. Protein content and amino acid composition of commercially available plant-based protein isolates. Amino Acids. (2018) 50:1685–95. doi: 10.1007/s00726-018-2640-5, PMID: 30167963 PMC6245118

[21] Devi S Varkey A Sheshshayee MS Preston T Kurpad AV. Measurement of protein digestibility in humans by a dual-tracer method. Am J Clin Nutr. (2018) 107:984–91. doi: 10.1093/ajcn/nqy062, PMID: 29771297 PMC6179135

[22] Bos C Mahé S Gaudichon C Benamouzig R Gausserès N Luengo C. Assessment of net postprandial protein utilization of 15 N-labelled milk nitrogen in human subjects. Br J Nutr. (1999) 81:221–6. doi: 10.1017/S0007114599000410 10434848

[23] Kashyap S Shivakumar N Sejian V Deutz NEP Preston T Sreeman S. Goat milk protein digestibility in relation to intestinal function. Am J Clin Nutr. (2021) 113:845–53. doi: 10.1093/ajcn/nqaa400, PMID: 33677496 PMC8023838

[24] van der Heijden I West S Monteyne AJ Finnigan TJA Abdelrahman DR Murton AJ. Algae ingestion increases resting and exercised Myofibrillar protein synthesis rates to a similar extent as Mycoprotein in young adults. Directory of graduate programs in nutritional sciences. (2023) 153:3406–17. doi: 10.1016/j.tjnut.2023.08.035, PMID: 37716611 PMC10739781

[25] van der Heijden I West S Monteyne AJ Finnigan TJA Abdelrahman DR Murton AJ. Ingestion of a variety of non-animal-derived dietary protein sources results in diverse postprandial plasma amino acid responses which differ between young and older adults. Br J Nutr. (2024) 131:1540–53. doi: 10.1017/S0007114524000163 38220222 PMC11043913

[26] Machado L Carvalho G Pereira RN. Effects of innovative processing methods on microalgae Cell Wall: prospects towards digestibility of protein-rich biomass. Biomass. (2022) 2:80–102. doi: 10.3390/biomass2020006

[27] van de Walle S Broucke K Baune MC Terjung N van Royen G Boukid F. Microalgae protein digestibility: how to crack open the black box? Crit Rev Food Sci Nutr. (2024) 64:7149–71. doi: 10.1080/10408398.2023.2181754, PMID: 38975868

[28] Safi C Ursu AV Laroche C Zebib B Merah O Pontalier PY. Aqueous extraction of proteins from microalgae: effect of different cell disruption methods. Algal Res. (2014) 3:61–5. doi: 10.1016/j.algal.2013.12.004

[29] Safi C Frances C Ursu AV Laroche C Pouzet C Vaca-Garcia C. Understanding the effect of cell disruption methods on the diffusion of *chlorella vulgaris* proteins and pigments in the aqueous phase. Algal Res. (2015) 8:61–8. doi: 10.1016/j.algal.2015.01.002

[30] Wang Y Tibbetts SM Berrue F McGinn PJ MacQuarrie SP Puttaswamy A. A rat study to evaluate the protein quality of three green microalgal species and the impact of mechanical cell wall disruption. Food Secur. (2020) 9:1531. doi: 10.3390/foods9111531, PMID: 33114413 PMC7694116

[31] Komaki H Yamashita M Niwa Y Tanaka Y Kamiya N Ando Y. The effect of processing of *Chlorella vulgaris*: K-5 on in vitro and in vivo digestibility in rats. Anim Feed Sci Technol. (1998) 70:363–6. doi: 10.1016/S0377-8401(97)00089-8

[32] Tessier R Calvez J Khodorova N Gaudichon C. Protein and amino acid digestibility of 15 N Spirulina in rats. Eur J Nutr. (2021) 60:2263–9. doi: 10.1007/s00394-020-02368-0 32870353

[33] Nuttall FQ Mooradian AD Gannon MC Billington C Krezowski P. Effect of protein ingestion on the glucose and insulin response to a standardized oral glucose load. Diabetes Care. (1984) 7:465–70. doi: 10.2337/diacare.7.5.465, PMID: 6389060

[34] Halton TL Hu FB. The effects of high protein diets on thermogenesis, satiety and weight loss: a critical review. J Am Coll Nutr. (2004) 23:373–85. doi: 10.1080/07315724.2004.10719381, PMID: 15466943

[35] Dehnavi Z Esfehani AJ Hajhoseini O Barghchi H Yazdi AG Khorasanchi Z. Postprandial effects of dietary protein source on metabolic responses, appetite, and arterial stiffness indices in overweight and obese men: the study protocol for a randomized crossover clinical trial. Trials. (2023) 24:415. doi: 10.1186/s13063-023-07374-1, PMID: 37337271 PMC10278248

[36] Westerterp-Plantenga MS Lemmens SG Westerterp KR. Dietary protein - its role in satiety, energetics, weight loss and health. Br J Nutr. (2012) 108:S105–12. doi: 10.1017/S0007114512002589 23107521

[37] Coelho MOC Monteyne AJ Kamalanathan ID Najdanovic-Visak V Finnigan TJA Stephens FB. High dietary nucleotide consumption for one week increases circulating uric acid concentrations but does not compromise metabolic health: a randomised controlled trial. Clin Nutr ESPEN. (2022) 49:40–52. doi: 10.1016/j.clnesp.2022.04.022, PMID: 35623844

[38] Calloway DH. (1969). “Safety of single-cell proteins as evaluated by human feeding trials at the University of California, Berkeley.” *16th meeting of the FAO/WHO/UNICEF Protein Advisory Group*.

[39] Sandgruber F Höger AL Kunze J Schenz B Griehl C Kiehntopf M. Impact of regular intake of microalgae on nutrient supply and cardiovascular risk factors: results from the NovAL intervention study. Nutrients. (2023) 15:1–21. doi: 10.3390/nu15071645 PMC10097350 37049486

[40] Finkel ZV Follows MJ Liefer JD Brown CM Benner I Irwin AJ. Phylogenetic diversity in the macromolecular composition of microalgae. PLoS One. (2016) 11:1–16. doi: 10.1371/journal.pone.0155977 PMC4882041 27228080

[41] Kaneko K Aoyagi Y Fukuuchi T Inazawa K Yamaoka N. Total purine and purine base content of common foodstuffs for facilitating nutritional therapy for gout and hyperuricemia. Biol Pharm Bull. (2014) 37:709–21. doi: 10.1248/bpb.b13-00967, PMID: 24553148

[42] Horstman AMH Huppertz T. Milk proteins: processing, gastric coagulation, amino acid availability and muscle protein synthesis. Crit Rev Food Sci Nutr. (2023) 63:10267–82. doi: 10.1080/10408398.2022.2078782, PMID: 35611879

[43] eChlorial. Chlorella echlorial: grown in glass tubes. (2024). Available from: https://www.echlorial.com/blog/chlorella-glass-tubes/ (Accessed June 21, 2021)

[44] Golden Green Organics. Organic chlorella powder. (2024). Available from: https://www.greensorganic.co.uk/products/organic-chlorella-powder?keyword=chlorella (Accessed June 21, 2021)

[45] Comerford KB Pasin G. Emerging evidence for the importance of dietary protein source on glucoregulatory markers and type 2 diabetes: different effects of dairy, meat, fish, egg, and plant protein foods. Nutrients. (2016) 8:446. doi: 10.3390/nu8080446 PMC4997361 27455320

[46] Lages M Barros R Moreira P Guarino MP. Metabolic effects of an Oral glucose tolerance test compared to the mixed meal tolerance tests: a narrative review. Nutrients. (2022) 14:1–15. doi: 10.3390/nu14102032 PMC9147413 35631171

[47] Abumrad NN Rabin D Diamond MP Lacy WW. Use of a heated superficial hand vein as an alternative site for the measurement of amino acid concentrations and for the study of glucose and alanine kinetics in man. Metabolism. (1981) 30:936–40. doi: 10.1016/0026-0495(81)90074-3, PMID: 7022111

[48] Flint A Raben A Blundell JE Astrup A. Reproducibility, power and validity of visual analogue scales in assessment of appetite sensations in single test meal studies. Int J Obes. (2000) 24:38–48. doi: 10.1038/sj.ijo.0801083, PMID: 10702749

[49] el Khoury D Vien S Sanchez-Hernandez D Kung B Wright A Goff HD. Increased milk protein content and whey-to-casein ratio in milk served with breakfast cereal reduce postprandial glycemia in healthy adults: an examination of mechanisms of action. Joint annual meeting abstracts. (2019) 102:6766–80. doi: 10.3168/jds.2019-16358, PMID: 31229285

[50] Weir JB. New methods for calculating metabolic rate with special reference to protein metabolism. J Physiol. (1949) 109:1–9. doi: 10.1113/jphysiol.1949.sp004363, PMID: 15394301 PMC1392602

[51] Hayes M. Measuring protein content method. Food Secur. (2020) 9:1–4. doi: 10.3390/foods9101340 PMC7597951 32977393

[52] West S Monteyne AJ Whelehan G van der Heijden I Abdelrahman DR Murton AJ. Ingestion of mycoprotein, pea protein, and their blend support comparable postexercise myofibrillar protein synthesis rates in resistance-trained individuals. Am J Physiol Endocrinol Metab. (2023) 325:E267–79. doi: 10.1152/ajpendo.00166.2023, PMID: 37529834 PMC10655824

[53] Nyakayiru J van Lieshout GAA Trommelen J van Kranenburg J Verdijk LB Bragt MCE. The glycation level of milk protein strongly modulates post-prandial lysine availability in humans. Br J Nutr. (2020) 123:545–52. doi: 10.1017/S0007114519002927, PMID: 31727194 PMC7015880

[54] Trommelen J Weijzen MEG van Kranenburg J Ganzevles RA Beelen M Verdijk LB. Casein protein processing strongly modulates post-prandial plasma amino acid responses in vivo in humans. Nutrients. (2020) 12:1–12. doi: 10.3390/nu12082299 PMC7468913 32751788

[55] Moore DR Robinson MJ Fry JL. Ingested protein dose response of muscle and albumin protein synthesis after resistance exercise in young men. Am J Clin Nutr. (2009) 89:161–8. doi: 10.3945/ajcn.2008.26401 19056590

[56] Witard OC Jackman SR Breen L Smith K Selby A Tipton KD. Myofibrillar muscle protein synthesis rates subsequent to a meal in response to increasing doses of whey protein at rest and after resistance exercise. Am J Clin Nutr. (2014) 99:86–95. doi: 10.3945/ajcn.112.055517 24257722

[57] Mitchell CJ McGregor R D’Souza R Thorstensen E Markworth J Fanning A. Consumption of milk protein or whey protein results in a similar increase in muscle protein synthesis in middle aged men. Nutrients. (2015) 7:8685–99. doi: 10.3390/nu7105420, PMID: 26506377 PMC4632440

[58] Gorissen SHM Trommelen J Kouw IWK Holwerda AM Pennings B Groen BBL. Protein type, protein dose, and age modulate dietary protein digestion and phenylalanine absorption kinetics and plasma phenylalanine availability in humans. J Nutr. (2020) 150:2041–50. doi: 10.1093/jn/nxaa024, PMID: 32069356 PMC7398787

[59] Dunlop MV Kilroe SP Bowtell JL Finnigan TJA Salmon DL Wall BT. Mycoprotein represents a bioavailable and insulinotropic non-animal-derived dietary protein source: a dose-response study. Br J Nutr. (2017) 118:673–85. doi: 10.1017/S0007114517002409, PMID: 29017627

[60] Gorissen SHM Witard OC. Characterising the muscle anabolic potential of dairy, meat and plant-based protein sources in older adults. Proc Nutr Soc. (2018) 77:20–31. doi: 10.1017/S002966511700194X, PMID: 28847314

[61] Dupont D Tomé D. Milk proteins: digestion and absorption in the gastrointestinal tract. Milk Proteins From Expr to Food. (2019) 701–14. doi: 10.1016/B978-0-12-815251-5.00020-7

[62] van Vliet S Burd NA van Loon LJC. The skeletal muscle anabolic response to plant- versus animal-based protein consumption. J Nutr. (2015) 145:1981–91. doi: 10.3945/jn.114.204305, PMID: 26224750

[63] Gorissen SHM Burd NA Hamer HM Gijsen AP Groen BB van Loon LJC. Carbohydrate coingestion delays dietary protein digestion and absorption but does not modulate postprandial muscle protein accretion. J Clin Endocrinol Metab. (2014) 99:2250–8. doi: 10.1210/jc.2013-3970 24628553

[64] Lin LP. Microstructure of spray-dried and freeze-dried microalgal powders. Food Struct Food Struct FOOD Microstruct. (1985) 4:341–8.

[65] Minic SL Stanic-Vucinic D Mihailovic J Krstic M Nikolic MR Cirkovic Velickovic T. Digestion by pepsin releases biologically active chromopeptides from C-phycocyanin, a blue-colored biliprotein of microalga Spirulina. J Proteome. (2016) 147:132–9. doi: 10.1016/j.jprot.2016.03.043, PMID: 27084687

[66] Safi C Zebib B Merah O Pontalier PY Vaca-Garcia C. Morphology, composition, production, processing and applications of *Chlorella vulgaris*: a review. Renew Sust Energ Rev. (2014) 35:265–78. doi: 10.1016/j.rser.2014.04.007

[67] Ding M Huang Z Jin Z Zhou C Wu J Zhao D. The effect of fat content in food matrix on the structure, rheological properties and digestive properties of protein. Food Hydrocoll. (2022) 126:107464. doi: 10.1016/j.foodhyd.2021.107464

[68] Zhang S de Vries S Gerrits WJJ. Quantifying the effects of dietary fibres on protein digestibility in pigs - a review. Anim Feed Sci Technol. (2024) 308:115864. doi: 10.1016/j.anifeedsci.2023.115864

[69] Blackburn N. Protein digestibility and absorption: effects of fibre, and the extent of individual variation. (1981). Available from: https://www.fao.org/4/M2836e/M2836e00.htm (Accessed July 21, 2021)

[70] Desideri G Castaldo G Lombardi A Mussap M Testa A Pontremoli R. Is it time to revise the normal range of serum uric acid levels? Eur Rev Med Pharmacol Sci. (2014) 18:1295–306. PMID: 24867507

[71] Waslien CI Calloway DH Margen S Costa F. Uric acid levels in men fed algae and yeast as protein sources. J Food Sci. (1970) 35:294–8. doi: 10.1111/j.1365-2621.1970.tb12166.x

