# Supplementary material for: Ingestion of ‘whole cell’ or ‘split cell’ Chlorella sp., Arthrospira sp., and milk protein show divergent postprandial plasma amino acid responses with similar postprandial blood glucose control in humans
Source: Front Nutr. 2024 Nov 14;11:1487778. doi: 10.3389/fnut.2024.1487778 (PMC11602285; doi:10.3389/fnut.2024.1487778)
Supplement: Supplementary file 1 [file Image_1.pdf]

Supplementary material 1

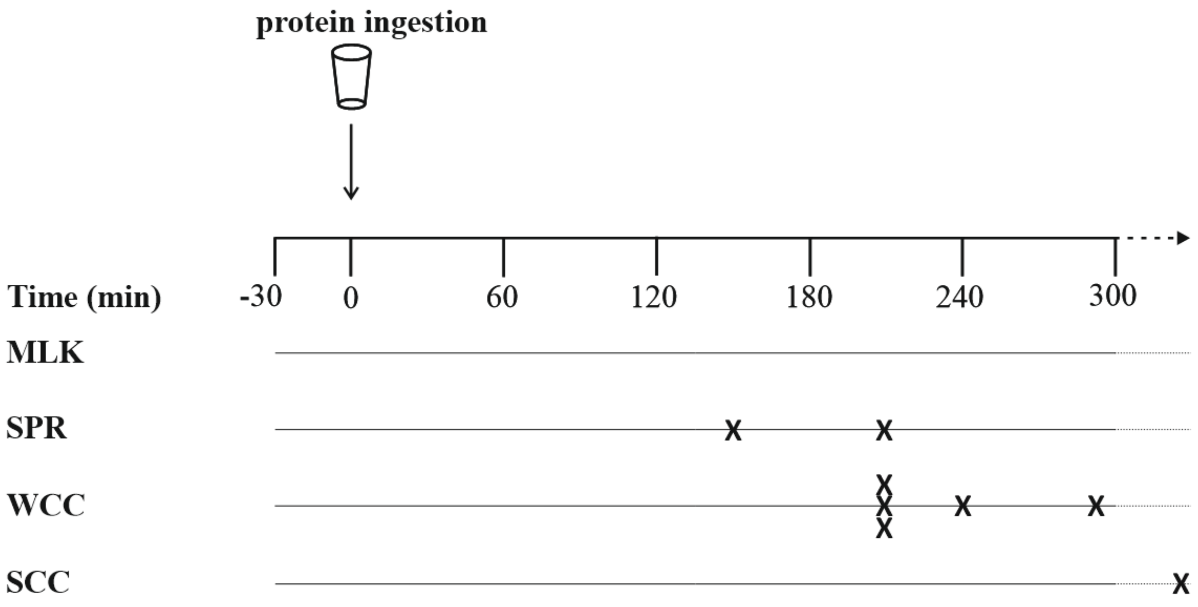

Supplementary figure 1. Timeline of adverse events (vomiting) marked by 'X', following the ingestion of 20 g milk protein (MLK), spirulina protein (SPR), whole cell chlorella protein (WCC), or split cell chlorella protein (SCC) in healthy young adults. Dotted line represents time after the 5 h postprandial period.
