# Supplementary material for: Ingestion of ‘whole cell’ or ‘split cell’ Chlorella sp., Arthrospira sp., and milk protein show divergent postprandial plasma amino acid responses with similar postprandial blood glucose control in humans
Source: Front Nutr. 2024 Nov 14;11:1487778. doi: 10.3389/fnut.2024.1487778 (PMC11602285; doi:10.3389/fnut.2024.1487778)
Supplement: Supplementary file 2 [file Image_2.pdf]

<u>Supplementary material 2</u>

Methods

Optical microscopy was used to visually examine chlorella cells and qualitatively determine the level of cell disruption between whole cell- (eChlorial®, France) and split cell- (Golden Greens Organic Ltd, UK) chlorella. Each sample was prepared by suspending 0.1 g chlorella powder, in 5 mL of water. Samples were inverted to ensure total suspension of powders. 20 µL of each sample were pipetted onto microscope slides and visualised using brightfield microscopy at 40 × magnification on the Leica DM IL LED (Leica Microsystems, Germany).

Results

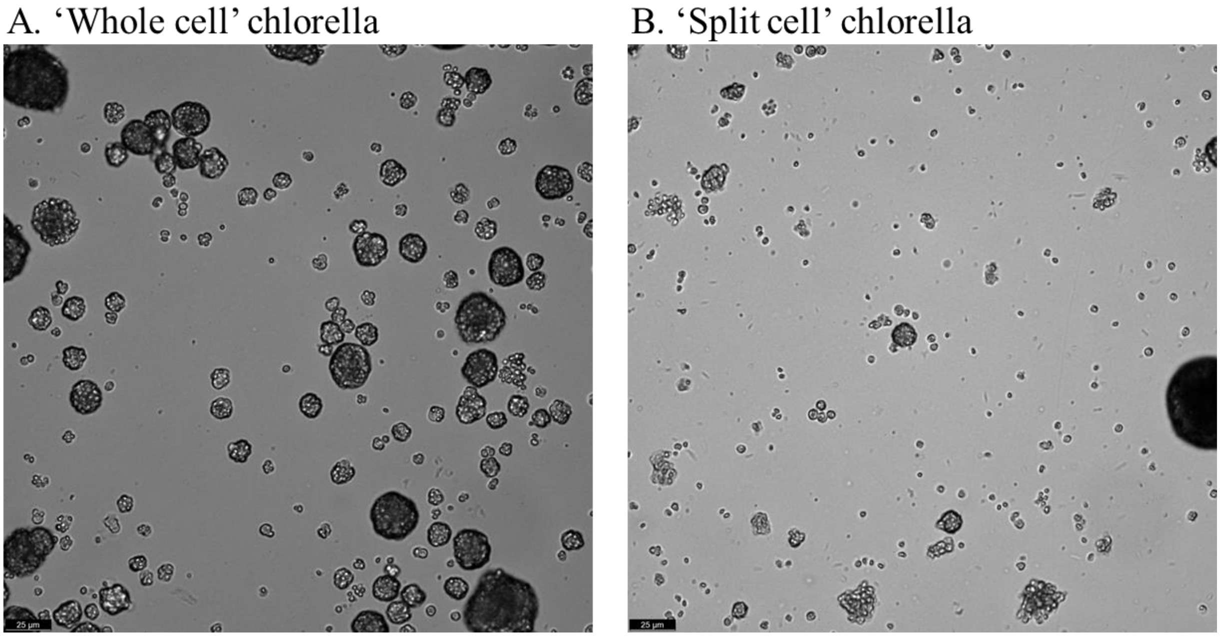

Supplementary figure 2. Optical bright field microscopy of dried **A.** 'whole cell' and **B.** 'split cell' chlorella, suspended in water. 40 × magnification.

Whole cell- and split cell- chlorella samples suspended in water are visualised in **Supplementary figure 2**. Observationally, there are fewer and smaller clusters of cells in the split cell- (**Supplementary figure 2A**) versus the whole cell- (**Supplementary figure 2B**) chlorella.
