# Supplementary material for: Ingestion of ‘whole cell’ or ‘split cell’ Chlorella sp., Arthrospira sp., and milk protein show divergent postprandial plasma amino acid responses with similar postprandial blood glucose control in humans
Source: Front Nutr. 2024 Nov 14;11:1487778. doi: 10.3389/fnut.2024.1487778 (PMC11602285; doi:10.3389/fnut.2024.1487778)
Supplement: Supplementary file 3 [file Image_3.pdf]

Supplementary material 3

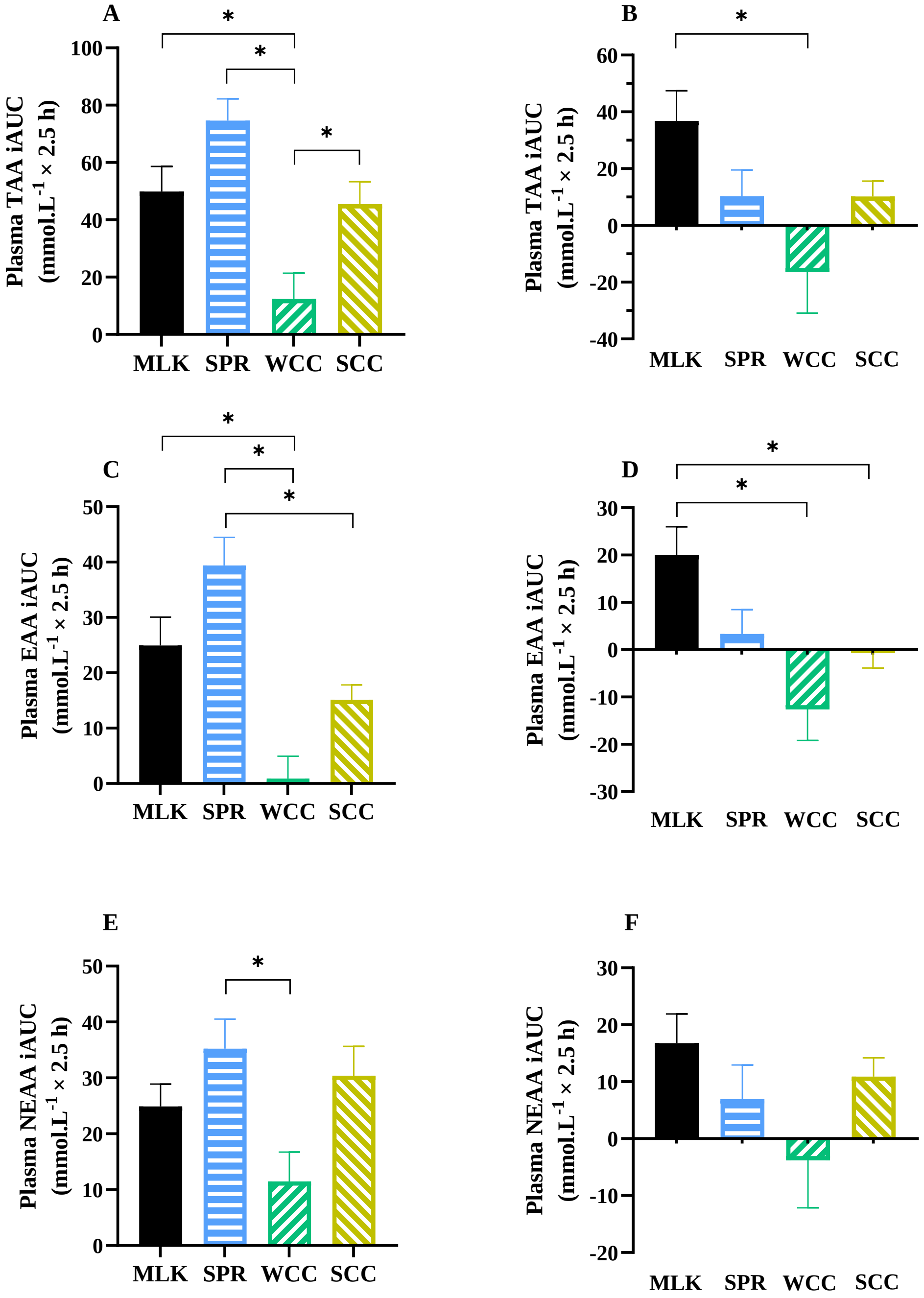

Supplementary figure 3. Incremental area under the curve of plasma total- (**A, B**), total essential- (**C, D**) and total non-essential- (**E, F**) amino acid concentrations following the ingestion of 20 g milk protein (MLK), spirulina protein (SPR), whole cell chlorella protein (WCC), or split cell chlorella protein (SCC) in healthy young adults (*n*=10). The 5 h postprandial period has been split into the first 0-2.5 h (**A, C, E**) and second 2.5-5 h (**B, D, F**) periods. Values are means, with their standard errors represented by vertical bars. Data were analysed for a main effect with a one-way ANOVA, and Tukey's multiple comparisons test applied to locate individual differences: * indicate values different from each other for MLK, SPR, WCC and SCC conditions.
