# Supplementary material for: Ingestion of ‘whole cell’ or ‘split cell’ Chlorella sp., Arthrospira sp., and milk protein show divergent postprandial plasma amino acid responses with similar postprandial blood glucose control in humans
Source: Front Nutr. 2024 Nov 14;11:1487778. doi: 10.3389/fnut.2024.1487778 (PMC11602285; doi:10.3389/fnut.2024.1487778)
Supplement: Supplementary file 4 [file Image_4.pdf]

Supplementary material 4

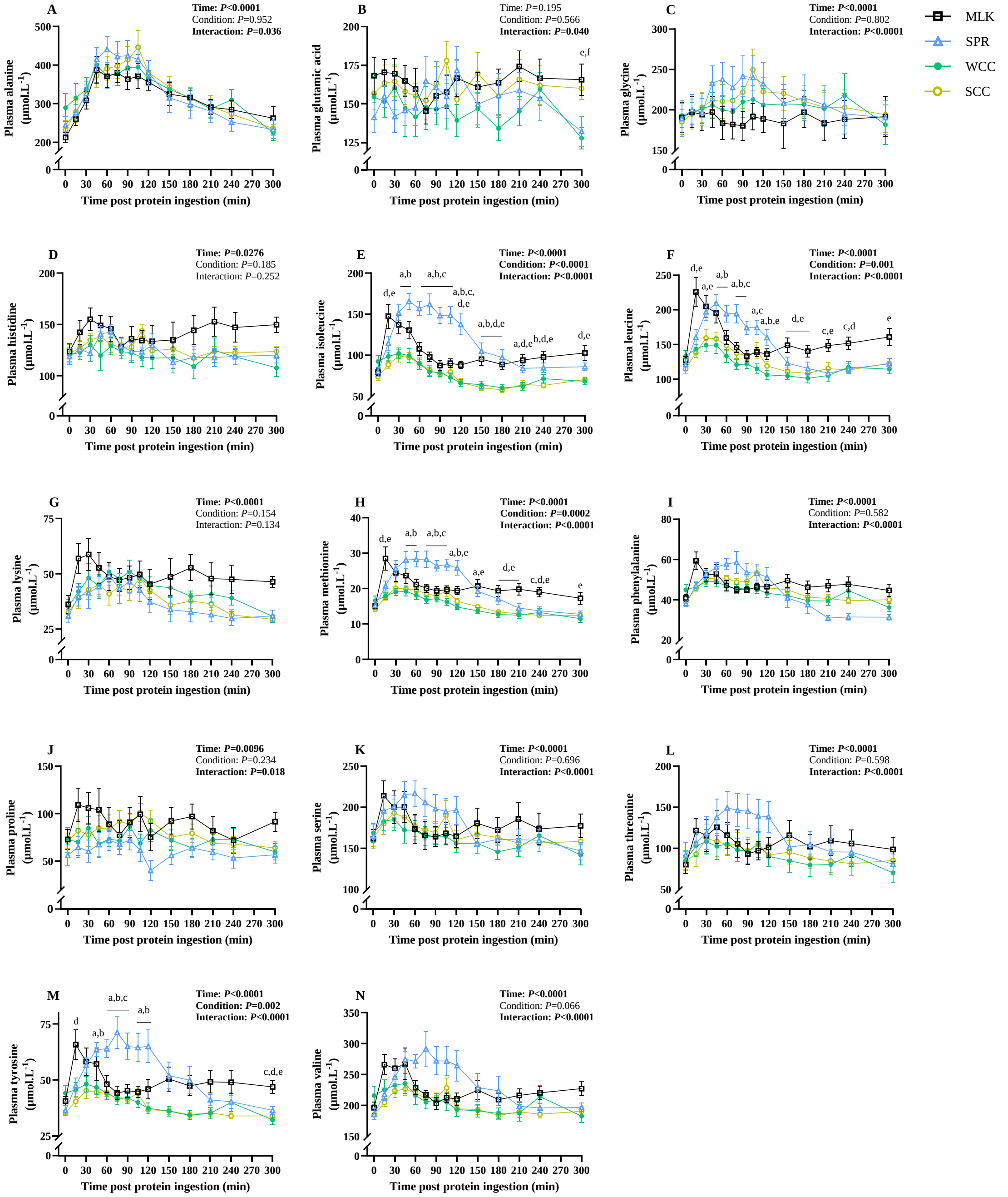

Supplementary Figure 4. Plasma total individual amino acid concentrations in the fasting state (*t*=0) and at regular intervals during a 5-h postprandial period following the ingestion of 20 g milk protein (□, MLK), spirulina protein (△, SPR), whole cell chlorella protein (●, WCC), or split cell chlorella protein (○, SCC) in healthy young adults (*n*=10). Values are means, with their standard errors represented by vertical bars. All conditions were statistically analysed with a two-way repeated measures ANOVA, significant *P* values are written in bold font. Tukey's multiple comparisons test was applied where appropriate to locate individual differences: a, b, c, d, e and f indicate values significantly different (*P*<0.05) between SPR vs WCC, SPR vs SCC, SPR vs MLK, MLK vs SCC, MLK vs WCC, and WCC vs SCC respectively.
