# Supplementary material for: Ingestion of ‘whole cell’ or ‘split cell’ Chlorella sp., Arthrospira sp., and milk protein show divergent postprandial plasma amino acid responses with similar postprandial blood glucose control in humans
Source: Front Nutr. 2024 Nov 14;11:1487778. doi: 10.3389/fnut.2024.1487778 (PMC11602285; doi:10.3389/fnut.2024.1487778)
Supplement: Supplementary file 6 [file Image_6.pdf]

Supplementary material 6

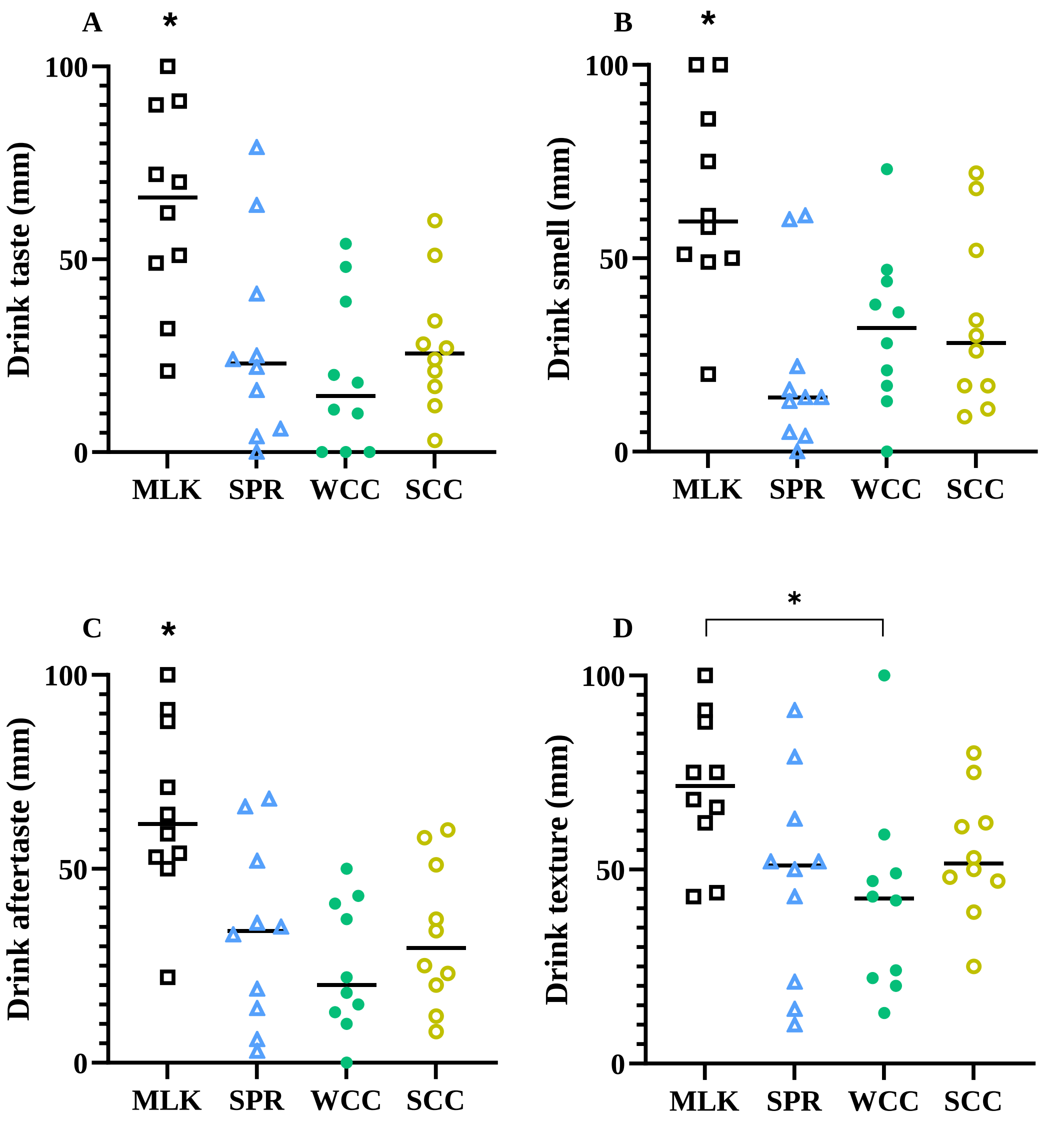

Supplementary figure 6. Palatability scores of **A.** drink taste, **B.** drink smell, **C.** drink aftertaste, **D.** drink texture were scored by healthy young adult participants (*n*=10) via visual analogue scales 5 minutes following the ingestion of 20 g milk protein (□, MLK), spirulina protein (Δ, SPR), whole cell chlorella protein (●, WCC), or split cell chlorella protein (○, SCC). All conditions were statistically analysed with a one-way ANOVA and Tukey's multiple comparison test applied to locate individual differences. * indicates significant difference between groups (P<0.05).
